# Development of a small molecule that corrects misfolding and increases secretion of Z α_1_‐antitrypsin

**DOI:** 10.15252/emmm.202013167

**Published:** 2021-01-29

**Authors:** David A Lomas, James A Irving, Christopher Arico‐Muendel, Svetlana Belyanskaya, Andrew Brewster, Murray Brown, Chun‐wa Chung, Hitesh Dave, Alexis Denis, Nerina Dodic, Anthony Dossang, Peter Eddershaw, Diana Klimaszewska, Imran Haq, Duncan S Holmes, Jonathan P Hutchinson, Alistair M Jagger, Toral Jakhria, Emilie Jigorel, John Liddle, Ken Lind, Stefan J Marciniak, Jeff Messer, Margaret Neu, Allison Olszewski, Adriana Ordonez, Riccardo Ronzoni, James Rowedder, Martin Rüdiger, Steve Skinner, Kathrine J Smith, Rebecca Terry, Lionel Trottet, Iain Uings, Steve Wilson, Zhengrong Zhu, Andrew C Pearce

**Affiliations:** ^1^ UCL Respiratory Rayne Institute University College London London UK; ^2^ GlaxoSmithKline Cambridge MA USA; ^3^ GlaxoSmithKline Stevenage UK; ^4^ GlaxoSmithKline Paris France; ^5^ Cambridge Institute for Medical Research Cambridgem UK

**Keywords:** emphysema, liver disease, protein misfolding, small molecule corrector, α_1_‐antitrypsin deficiency, Genetics, Gene Therapy & Genetic Disease, Chemical Biology

## Abstract

Severe α_1_‐antitrypsin deficiency results from the Z allele (Glu342Lys) that causes the accumulation of homopolymers of mutant α_1_‐antitrypsin within the endoplasmic reticulum of hepatocytes in association with liver disease. We have used a DNA‐encoded chemical library to undertake a high‐throughput screen to identify small molecules that bind to, and stabilise Z α_1_‐antitrypsin. The lead compound blocks Z α_1_‐antitrypsin polymerisation *in vitro*, reduces intracellular polymerisation and increases the secretion of Z α_1_‐antitrypsin threefold in an iPSC model of disease. Crystallographic and biophysical analyses demonstrate that GSK716 and related molecules bind to a cryptic binding pocket, negate the local effects of the Z mutation and stabilise the bound state against progression along the polymerisation pathway. Oral dosing of transgenic mice at 100 mg/kg three times a day for 20 days increased the secretion of Z α_1_‐antitrypsin into the plasma by sevenfold. There was no observable clearance of hepatic inclusions with respect to controls over the same time period. This study provides proof of principle that “mutation ameliorating” small molecules can block the aberrant polymerisation that underlies Z α_1_‐antitrypsin deficiency.

## Graphical abstract

A chemistry campaign has developed a small molecule that stabilises the severe Z deficiency mutant of α1‐antitrypsin. The lead compound binds to a cryptic pocket and blocks the conformational change and pathological polymerisation that underlie α1‐antitrypsin deficiency.

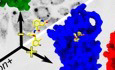

**The paper explained**

**Problem**

Intracellular protein aggregation can result in “gain‐of‐function” cell toxicity. It has proved challenging to develop small molecules that can stabilise intracellular mutant proteins, prevent self‐aggregation and so ameliorate disease. Severe α_1_‐antitrypsin deficiency results largely from the Z allele (Glu342Lys) that causes the accumulation of homopolymers of mutant α_1_‐antitrypsin within the endoplasmic reticulum of hepatocytes in association with liver disease.

**Results**

We have undertaken a medicinal chemistry campaign to develop an orally bioavailable small molecule that binds to intra‐endoplasmic reticulum mutant Z α_1_‐antitrypsin, corrects the folding defect and increases secretion in a transgenic model of disease.

**Impact**

This study reports the successful targeting of an aggregation‐prone mutant in order to prevent the intracellular polymerisation and accumulation of α_1_‐antitrypsin that underlies α_1_‐antitrypsin deficiency. It demonstrates that “mutation ameliorating” small molecules can block the aberrant polymerisation that underlies Z α_1_‐antitrypsin deficiency.

## Introduction

Alpha‐1 antitrypsin deficiency affects 1 in 2,000 people of Northern European descent, leading to liver and lung diseases (Lomas *et al*, 2016). Ninety‐five per cent of severe deficiency results from the “Z” allele (Glu342Lys) that perturbs the folding of α_1_‐antitrypsin resulting in the secretion of only 15% of the mature protein. The remaining protein is retained within the cell by persistent binding to molecular chaperones (Wu *et al*, 2003) and then either degraded via the ERAD‐proteasome pathway (Le *et al*, 1992; Qu *et al*, 1996; Teckman *et al*, 2001) or folded into ordered polymers that may be cleared by autophagy (Teckman *et al*, 2004) or accumulate within the endoplasmic reticulum (ER) of hepatocytes (Lomas *et al*, 1992). The accumulation of polymers causes neonatal hepatitis, cirrhosis and hepatocellular carcinoma, and can sensitise the liver to damage from environmental insults such as alcohol, fat or viral hepatitis (Ordóñez *et al*, 2013; Strnad *et al*, 2019). The consequent deficiency of α_1_‐antitrypsin within the circulation results in insufficient protection of the lungs from neutrophil elastase, leading to early‐onset emphysema (Lomas *et al*, 2016).

The Z mutation lies at the head of strand 5 of β‐sheet A of α_1_‐antitrypsin. It perturbs the local environment, allowing population of an unstable intermediate that we have termed M* (Dafforn *et al*, 1999) in which β‐sheet A opens and the upper part of helix F unwinds (Gooptu *et al*, 2000; Nyon *et al*, 2012). Polymerisation from this state involves insertion of the RCL into β‐sheet A with a domain‐swap of the C‐terminal region providing the inter‐subunit linkage (Huang *et al*, 2016; Faull *et al*, 2020; Laffranchi *et al*, 2020). The resulting polymer is deposited within hepatocytes.

The aim of our work was to develop a small molecule corrector of Z α_1_‐antitrypsin folding that was able to block the formation of polymers within the ER of hepatocytes and that was suitable for oral dosing as a potential treatment for α_1_‐antitrypsin deficiency. To achieve this, we needed to overcome a number of challenges: (i) the drug target is a highly mobile folding intermediate located in the ER; (ii) disparity in the size of the interface between a small molecule and the large protein–protein interaction that it is designed to block; (iii) oral dosing greatly restricts suitable chemical space; (iv) as a non‐classical drug target, small molecule binders may well not be well‐represented in compound screening libraries; and (v) the relatively high concentration of circulating monomeric Z α_1_‐antitrypsin (~ 5 μM), even in individuals with severe plasma deficiency, represents a high‐affinity sink for compound, restricting its access to the target in the hepatocyte and requiring high total blood concentrations of drug to achieve sufficient free drug concentration and target engagement in the liver.

## Results

### Identification of GSK716 through encoded library technology screening, structure‐guided drug design and cellular profiling

**Figure 1:**
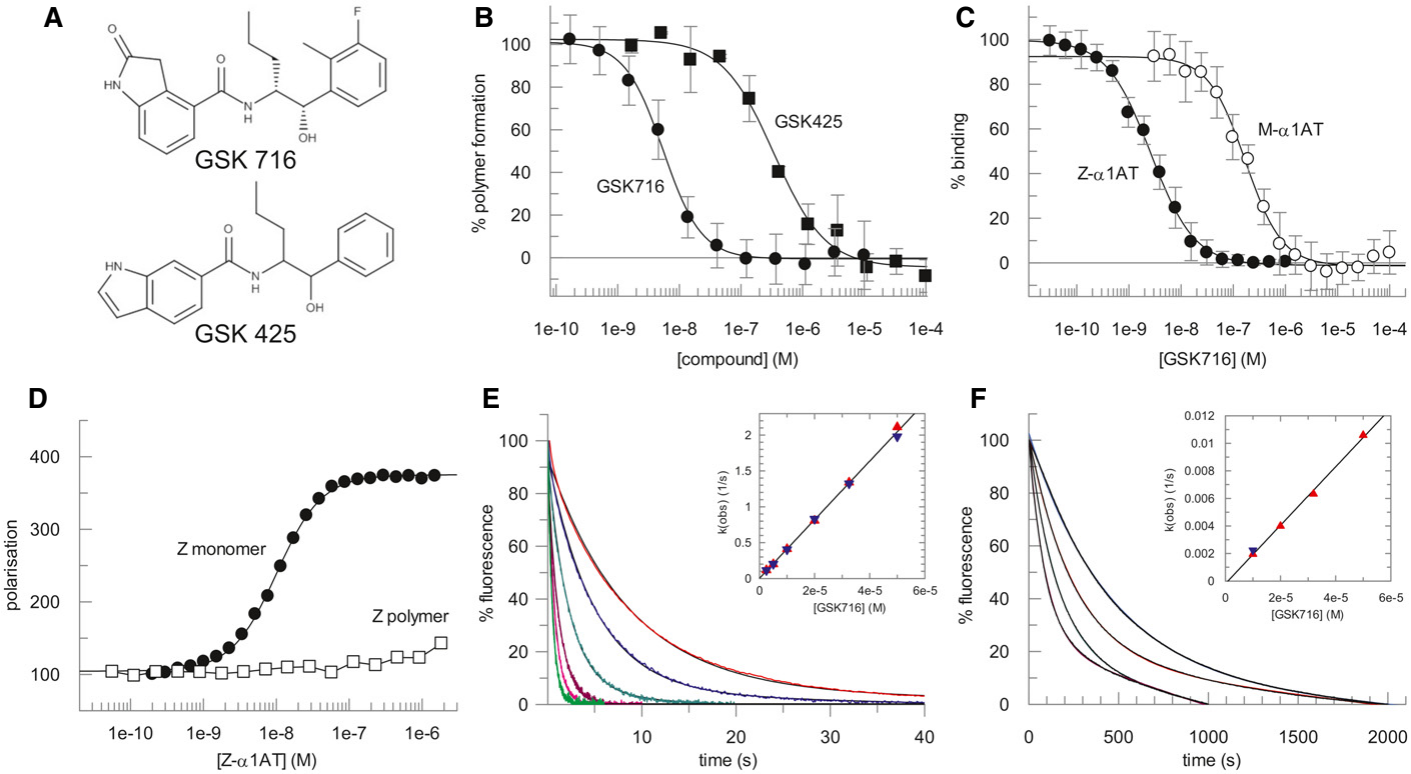
Characteristics of the lead series of chiral hydroxy‐carboxamides identified from the ELT screen The structure of GSK425, identified from the ELT screen, and the derived compound GSK716 obtained through a structure‐based design pipeline.The degree of polymerisation of Z α_1_‐antitrypsin after 72 h at 37°C, as determined by an end‐point immunoassay using the 2C1 monoclonal antibody, in varying concentrations of compound (shown in panel A). Modification of the phenyl and indole heterocycle of GSK425 (pIC50 6.5) resulted in an ~ 100‐fold increase in potency and the discovery of the 2‐oxindole GSK716 (pIC50 8.3). Data presented as mean ± SD, *n* = 2 (GSK425) and *n* = 25 (GSK716).GSK716 binds to Z α_1_‐antitrypsin with a high‐affinity mean pKD of 8.5 ± 0.12 (*n* = 18) as determined by a competition binding assay with a fluorescently labelled derivative. There was a 50‐fold lower affinity for plasma‐purified wild‐type M α_1_‐antitrypsin, with a mean pKD of 6.8 ± 0.18 (*n* = 10). Data presented as mean ± SD.The compound bound to monomeric but not polymeric Z α_1_‐antitrypsin (Z α_1_‐AT) as reported by fluorescence polarisation of an Alexa‐488‐labelled variant of GSK716.Representative curves reporting the interaction of different concentrations of GSK716 with Z α_1_‐antitrypsin based on changes in intrinsic tryptophan fluorescence. Based on the concentration dependence (*inset*), the second‐order rate constant of association was found to be 4.1 × 10^4^ M^−1^ s^−1^.The association of GSK716 with M α_1_‐antitrypsin, giving a second‐order rate constant of 2.1 × 10^2^ M^−1^ s^−1^.

Z α_1_‐antitrypsin is a conformationally dynamic molecule (Lomas *et al*, 1992; Knaupp *et al*, 2010) that represents a non‐classical target for drug discovery. A cell‐free assay approach to hit finding was undertaken so as not to miss compounds that bind α_1_‐antitrypsin and block polymerisation but lack the molecular properties to cross cell membranes. This comprised the following: (i) an encoded library technology (ELT) screen (Goodnow *et al*, 2017) of a library with a nominal diversity of 2 × 10^12^ unique components to identify binders to Z α_1_‐antitrypsin and (ii) a high‐throughput screen (HTS) of the GSK compound collection (~ 1.7 million compounds) for small molecules that could block polymerisation of Z α_1_‐antitrypsin. In both screening approaches, glycosylated Z α_1_‐antitrypsin, purified from the plasma of Z α_1_‐antitrypsin homozygotes (Lomas *et al*, 1993), was used since this represents the disease‐relevant human pathophysiological drug target that populates an intermediate on the polymerisation pathway (Knaupp *et al*, 2010; Irving *et al*, 2015). ELT selections were performed by incubating Z α_1_‐antitrypsin with DNA‐encoded compound libraries for 1 h at 4 and 37°C for three rounds of selection with subsequent capture of Z α_1_‐antitrypsin using α_1_‐antitrypsin select resin (GE Healthcare). A variation on this protocol using pre‐immobilised Z α_1_‐antitrypsin was also used for library selections. In the HTS assay, polymerisation of purified Z α_1_‐antitrypsin was induced by incubation at 37°C for 72 h in the presence of test compounds, with end‐point quantification of polymers performed using the polymer‐specific monoclonal antibody, 2C1 (Miranda *et al*, 2010) in a TR‐FRET‐based immunoassay. A number of small molecules that could block polymerisation of Z α_1_‐antitrypsin were obtained through the HTS but none progressed beyond the early lead optimisation stage. However, a single lead series of chiral hydroxy‐carboxamides (GSK425) was identified from the ELT screen that also demonstrated functional activity at blocking polymerisation in the TR‐FRET immunoassay (pIC50 6.5; Fig 1A and B).

Optimisation of this initial hit followed a structure‐based design approach, exploiting knowledge from iterative crystal structures of small molecule ligands complexed with α_1_‐antitrypsin. The central hydroxy carboxamide and propyl chain were found to be critical for binding to Z α_1_‐antitrypsin and hence further medicinal chemistry development focussed on modification of the phenyl and indole heterocycle. This resulted in an ~ 100‐fold increase in potency and the discovery of the 2‐oxindole GSK716 (pIC50 8.3; Fig 1A and B).

### GSK716 is a potent inhibitor of polymerisation *in vitro* and in cell models of disease

GSK716 binds to Z α_1_‐antitrypsin with a high‐affinity mean pKD 8.5 ± 0.12 (*n* = 18) as determined by a competition binding assay with a fluorescently labelled derivative (Fig 1C). The binding demonstrates selectivity with a 50‐fold lower affinity for plasma‐purified wild‐type M α_1_‐antitrypsin at mean pKD 6.8 ± 0.18 (*n* = 10; Fig 1C). The shape of the curves and native mass spectrometry (not shown) are consistent with a single high‐affinity compound binding site. No binding of the fluorescent derivative to polymers of Z α_1_‐antitrypsin was observed, indicating conformational selectivity for the monomeric protein (Fig 1D). The rate of interaction of the compound with the target was monitored through changes in intrinsic tryptophan fluorescence (Dafforn *et al*, 1999); this property was used to determine the second‐order association rate constants for GSK716 binding to Z (4.1 × 10^4^ M^−1^ s^−1^) and M α_1_‐antitrypsin (2.1 × 10^2^ M^−1^ s^−1^; Fig 1E and F). From the association rate constants and the affinity values, first‐order dissociation rate constants were calculated and found to be of the same order of magnitude for Z (6.1 × 10^−5^ s^−1^) and M α_1_‐antitrypsin (1.6 × 10^−5^ s^−1^). Therefore, the selectivity of the compound for Z over M α_1_‐antitrypsin is dominated by the difference in the rate of association rather than dissociation.

**Figure 2:**
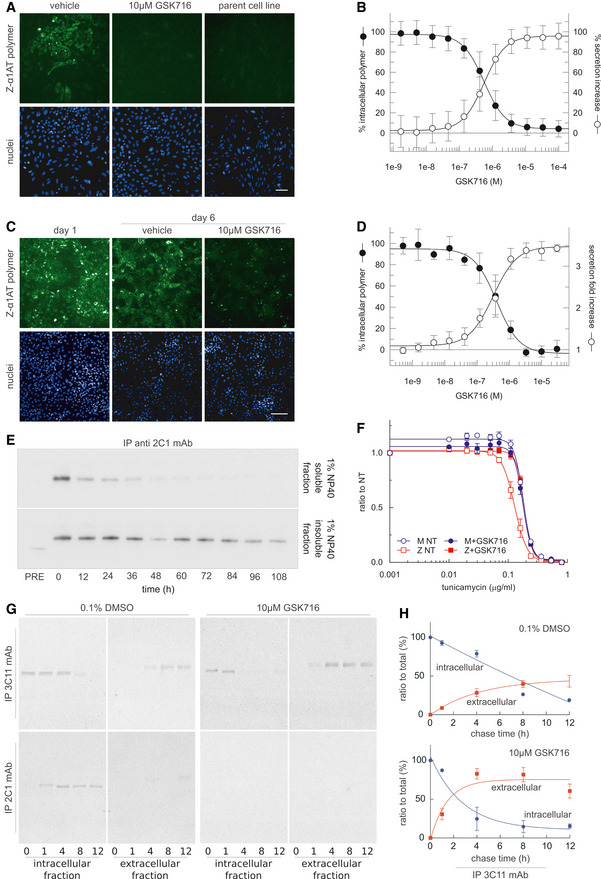
GSK716 inhibits polymerisation of Z α_1_‐antitrypsin in cell models of disease AGSK716 was added to CHO‐TET‐ON‐Z‐A1AT cells (Ordóñez *et al*, 2013) with simultaneous induction of Z α_1_‐antitrypsin expression using doxycycline, and polymer load was quantified with the 2C1 monoclonal antibody that is specific to pathological polymers of α_1_‐antitrypsin (Miranda *et al*, 2010). The parent cell line that did not express Z α_1_‐antitrypsin provided a negative control. GSK716 completely prevented intracellular polymer formation. Scale bar: 50 µm.BQuantification of immunostained CHO‐TET‐ON‐Z‐A1AT cells showed that GSK716 reduced intracellular polymer formation and increased the secretion of Z α_1_‐antitrypsin in a dose‐dependent manner with similar potencies. Data were normalised to vehicle and a control compound from the GSK716 series at saturating concentration. Data presented as mean ± SD, *n* = 61 (secretion) and *n* = 67 (polymer inhibition).C, DGSK716 was (C) administered to iPSC‐derived‐hepatocytes and (D) inhibited polymerisation and increased secretion with a similar potency. It induced an approximately threefold increase in secreted levels of Z α_1_‐antitrypsin compared with vehicle control. This was apparent even after polymers had been allowed to form. Scale bar: 100 µm. Data presented as mean ± SD, *n* = 8 (secretion) and *n* = 6 (polymer inhibition).ECHO tetracycline‐inducible cells expressing Z α_1_‐antitrypsin were induced with 0.5 μg/ml doxycycline for 48 h and treated with 10 μM GSK716. Cells were then lysed in 1% v/v NP‐40 buffer at different time points (0, 12, 24, 36, 48, 60, 72, 84, 96 and 108 h). For every time point, NP‐40‐soluble and NP‐40‐insoluble fractions were separated and immunoprecipitated with the 2C1 mAb and resolved by 4–12% w/v SDS–PAGE and α_1_‐antitrypsin detected by immunoblotting. PRE indicates cells pre‐treated for 48 h with 10 μM GSK716 and induced for the same time with 0.5 μg/ml doxycycline. The rate of clearance of soluble and insoluble polymer is shown.FCHO‐inducible cells expressing either wild‐type M or Z α_1_‐antitrypsin were induced with 0.5 μg/ml doxycycline and treated with 10 μM GSK716 or with 0.1% DMSO vehicle (NT, not treated). After induction for 48 h, cells were treated with various doses of tunicamycin for 36 h. Cell viability was measured by Cell Counting Kit‐8. The results are shown as mean ± SEM, *n* = 4.GCHO‐K1 Tet‐On cells expressing Z α_1_‐antitrypsin were induced with doxycycline (0.5 μg/ml) for 48 h. Cells were incubated with 10 μM GSK716 (or 0.1% v/v DMSO for the control) during the induction. Culture media containing either the experimental compound or DMSO were changed every 24 h. After the induction, cells were labelled for 10 min with ^35^S Met/Cys and chased at the indicated times. Culture media were collected and cells lysed in 1% v/v NP‐40 buffer. Intracellular fractions and culture media from cells expressing Z α_1_‐antitrypsin were immunoprecipitated either with a mAb against total α_1_‐antitrypsin (3C11) or with a polymer‐specific mAb (2C1). Samples were resolved by 4–12% w/v acrylamide SDS–PAGE and detected by autoradiography.HThe graphs show the effect of GSK716 on intracellular and extracellular Z α_1_‐antitrypsin (mean ± SEM, *n* = 2).

The ability of GSK716 to block Z α_1_‐antitrypsin polymerisation in the ER during folding was assessed by adding GSK716 to CHO‐TET‐ON‐Z‐A1AT cells (Ordóñez *et al*, 2013) with simultaneous induction of Z α_1_‐antitrypsin expression using doxycycline. In comparison with controls, GSK716 completely blocked the intracellular formation of Z α_1_‐antitrypsin polymers, as measured by staining with the 2C1 anti‐Z α_1_‐antitrypsin polymer monoclonal antibody (mean pIC50 = 6.3 ± 0.23; *n* = 71; Fig 2A and B). It also increased the secretion of Z α_1_‐antitrypsin (mean pEC50 6.2 ± 0.23; *n* = 74; Fig 2B). Similar potency between the effects on secretion and polymerisation was observed throughout members of the lead series supporting the hypothesis that these effects are caused by the same pharmacological mode of action. GSK716 had a similar effect on the secretion and polymerisation of constitutively expressed Z α_1_‐antitrypsin in iPSC‐derived human hepatocytes with the ZZ α_1_‐antitrypsin genotype (Yusa *et al*, 2011). It inhibited polymerisation and increased secretion with a mean pIC50 of 6.4 ± 0.45 (*n* = 16) and mean pEC50 of 6.5 ± 0.37 (*n* = 14), respectively, inducing an approximately threefold increase in secreted levels of Z α_1_‐antitrypsin (Fig 2C and D). GSK716 treatment reduced the levels of intracellular Z α_1_‐antitrypsin polymer compared with cells assessed before compound addition (Fig 2C), demonstrating that polymers can be cleared over the time course of the experiment, and that accumulation of polymers is reversible in ZZ‐iPSC hepatocytes.

The pre‐treatment of CHO cells induced to express Z α_1_‐antitrypsin with GSK716 significantly reduced the formation of soluble and insoluble polymers (Fig 2E; compare PRE with time 0). To investigate the ability of GSK716 to protect cells from sensitisation to a secondary insult, Z α_1_‐antitrypsin expression was induced in CHO‐TET‐ON‐Z‐A1AT cells in the presence or absence of 10 μM GSK716 before exposure to increasing concentrations of the ER stressor tunicamycin (Ordóñez *et al*, 2013). Cells expressing wild‐type M α_1_‐antitrypsin were less susceptible to tunicamycin toxicity than cells expressing Z α_1_‐antitrypsin in a cell viability assay (Fig 2F). GSK716 restored sensitivity of Z α_1_‐antitrypsin expressing cells to that of the wild‐type control cells. The effect of GSK716 on Z α_1_‐antitrypsin was confirmed in pulse‐chase experiments (Fig 2G and H).

These data collectively show that the small molecule completely blocks the intracellular polymerisation of Z α_1_‐antitrypsin and increases secretion of the monomeric protein.

### GSK716 binds to a novel cryptic binding site

**Figure 3:**
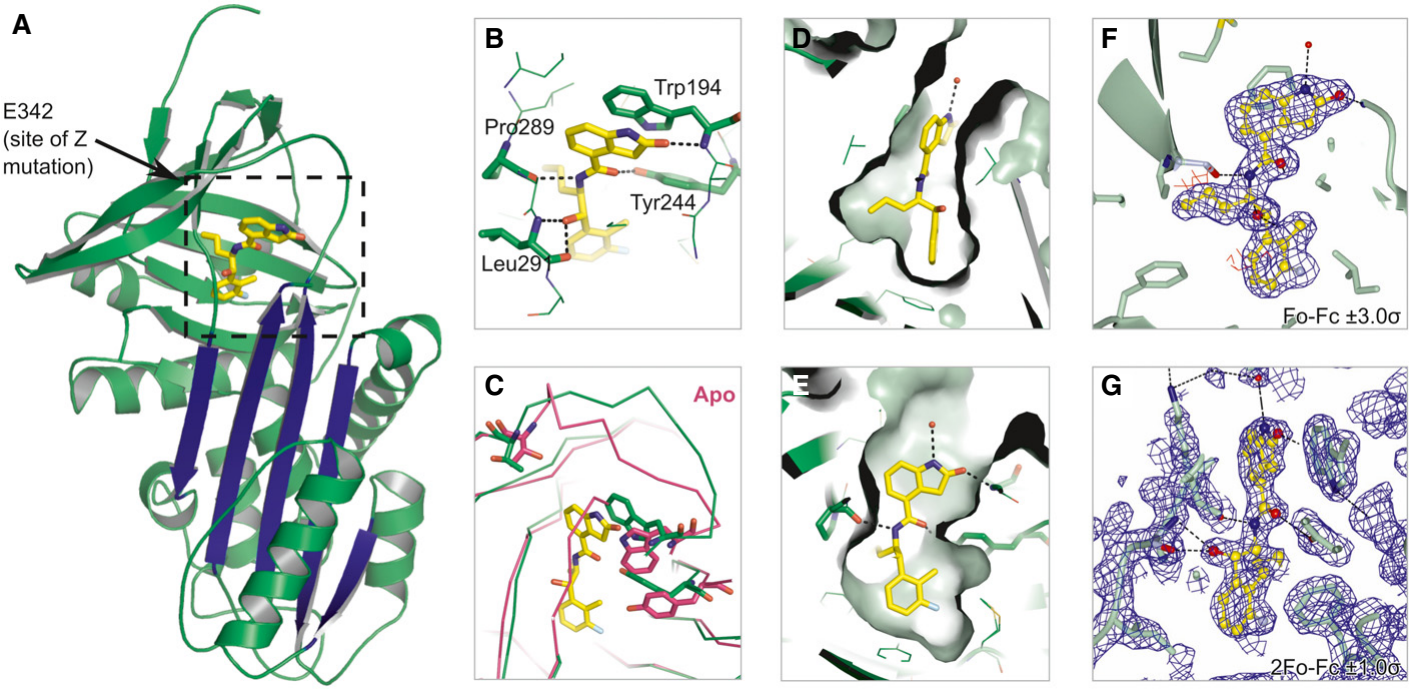
GSK716 binds to a novel cryptic binding site Cartoon representation of α_1_‐antitrypsin with GSK716 shown in yellow stick format. The five‐stranded β‐A sheet is in blue.Interactions of GSK716 within the cryptic site with key residues shown in stick format and hydrogen bonds in black dashed lines.Overlay of ribbon representation of protein chains of GSK716 complex (green) with apoprotein (purple; PDB id 2QUG (Pearce *et al*, 2008)) showing the altered position of Trp194 (stick format) that induces a change in conformation of the Gly192‐to‐Thr203 loop and re‐orientation of Tyr244 (stick format).Surface representation of α_1_‐antitrypsin to show propyl and phenyl pocket.Surface representation of α_1_‐antitrypsin to show protein‐ligand complement of the substituted phenyl and 2‐oxindole rings.Fo‐Fc omit density difference map for the ligand contoured at 3 sigma (red denotes negative, and blue denotes positive difference).Representative 2Fo‐Fc electron density map contoured at 1.0 sigma around the ligand.

A high‐resolution crystal structure of α_1_‐antitrypsin complexed with the lead compound GSK716 was generated by soaking compound into apo α_1_‐antitrypsin crystals (Table 1). The structure reveals that interaction with the compound induces the formation of a cryptic binding site that is not evident in apo structures, at the top of β‐sheet A behind strand 5. This region is referred to as the “breach” as it is the point at which the reactive centre loop first inserts during protease inhibition (Whisstock *et al*, 2000), and includes the site of the Z (Glu342Lys) mutation (Fig 3A). The structure reveals that the 2‐oxindole ring of GSK716 stacks with the side chain of Trp194 whilst the carbonyl group forms a hydrogen bond with the mainchain Trp194 (Fig 3B). Trp194 adopts a new position due to rearrangement of residues Gly192 to Thr203 consistent with the change in intrinsic tryptophan fluorescence induced by binding (Fig 3C). The phenyl ring and the propyl chain occupy two highly hydrophobic pockets (Fig 3D–G). Hydrogen bonds are formed between the GSK716 hydroxyl group and the Leu291 backbone, the amide nitrogen hydrogen and the backbone carbonyl oxygen of Pro289, and between the amide carbonyl and the Tyr 244 hydroxyl group (Fig 3B). This causes displacement of residues Thr339 to Ser 359 of strand 5A relative to the apoprotein. Few changes are seen outside of these regions.

**Table 1:** Data collection and refinement statistics.

	7AEL
Temperature	100K
Wavelength	0.9763
Resolution range	55.05–1.76 (1.823–1.76)
Space group	C 1 2 1
Unit cell	113.95 39.59 90.52 90 104.96 90
Total reflections	127,818 (12,581)
Unique reflections	38,772 (3,853)
Multiplicity	3.3 (3.3)
Completeness (%)	99.2 (99.2)
Mean *I*/sigma (*I*)	19.61 (2.19)
Wilson *B*‐factor	32.95
R‐merge	0.02941 (0.5007)
R‐meas	0.03523 (0.5991)
CC1/2	0.999 (0.805)
CC*	1 (0.944)
Reflections used in refinement	38,771 (3,853)
Reflections used for R‐free	1,908 (178)
R‐work	0.1969 (0.3065)
R‐free	0.2259 (0.3204)
CC(work)	0.958 (0.739)
CC(free)	0.943 (0.699)
Number of non‐hydrogen atoms	3,189
Macromolecules	2,868
Ligands	32
Protein residues	357
RMS (bonds)	0.005
RMS (angles)	1.02
Ramachandran favoured (%)	99
Ramachandran allowed (%)	1.1
Ramachandran outliers (%)	0
Rotamer outliers (%)	0.62
Clashscore	0.52
Average *B*‐factor	47.79
Macromolecules	46.77
Ligands	36.49
Solvent	59.23

Statistics for the highest resolution shell are shown in parentheses.

### GSK716 interferes with the transition through the polymerisation‐prone intermediate M* by stabilising β‐sheet A

**Figure 4:**
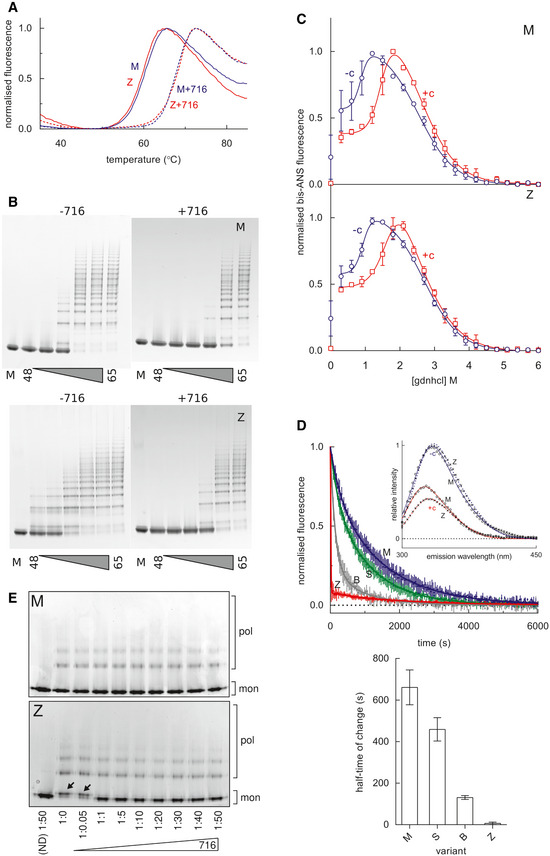
Stabilisation of α_1_‐antitrypsin by GSK716 A SYPRO orange‐based thermal stability assay, which reports the transition of M α_1_‐antitrypsin and Z α_1_‐antitrypsin from the native to an intermediate state over a 1°C/min thermal ramp, in the presence and absence of 50µM GSK716.M (above) and Z (below) α_1_‐antitrypsin, at a concentration of 0.2 mg/ml in PBS + 5% v/v glycerol, were heated at a range of temperatures between 48 and 65°C for 4 h in the presence and absence of GSK716, as indicated. The oligomerisation state was determined by non‐denaturing PAGE electrophoresis; the lane denoted “M” contains the unheated monomeric control.M α_1_‐antitrypsin and Z α_1_‐antitrypsin were subjected to equilibrium unfolding into different concentrations of guanidine hydrochloride (gdnhcl) in the presence and absence of GSK716, with bis‐ANS dye added to report the presence of the unfolded intermediate. The normalised fluorescence intensity data were fitted with an equation describing a three‐state unfolding curve. Values shown are the mean of three independent experiments, and the error bars represent ± SEM.
*Top panel*, the association of GSK716 induced a marked quenching and blue‐shift of the α_1_‐antitrypsin intrinsic tryptophan fluorescence spectrum with respect to unbound protein (inset graph). The association of 10 µM GSK716 with four α_1_‐antitrypsin variants that vary in their propensity to polymerise, ranging from inert (M), to mild (S), moderate (Baghdad denoted “B”) and severe (Z). Representative progress curves of the change in intrinsic tryptophan fluorescence at 330 nm for 0.2 mg/ml protein are shown. *Bottom panel*, the half‐time of association calculated from three such independent experiments (error bars are ± SEM) show a correspondence with the polymerisation propensity of the four variants. The change in Z α_1_‐antitrypsin fluorescence was faster than the dead‐time of the apparatus (~ 10 s), and scaling was with reference to unbound intensity.Non‐denaturing PAGE characterisation of the conformational state of M α_1_‐antitrypsin and Z α_1_‐antitrypsin, after rapidly refolding by snap dilution from 6M urea in the presence or absence of GSK716 at the molar ratios indicated. The migration of polymers (pol) and monomers (mon) is shown. Arrows indicate the misfolded by‐product M* arising at sub‐stoichiometric concentrations of compound.

Polymerisation of α_1_‐antitrypsin involves transition through a transient intermediate state known as M*, that is readily populated by the Z variant (Dafforn *et al*, 1999; Knaupp *et al*, 2010). The M* conformational ensemble appears to be a distinct species between the native state conformation and that of the final polymer. One of the hallmarks of M* is its recognition by environment‐sensitive fluorescent reporter dyes. Thermal shift assays that make use of the dye SYPRO Orange report the stability of the protein native state against heat‐induced unfolding. Experiments, performed using different temperature gradients in the presence and absence of 50μM GSK716, demonstrated a marked increase in the transition midpoint temperature (Fig 4A), consistent with the stabilisation of either or both of the ground‐ and M*‐states of α_1_‐antitrypsin (Irving *et al*, 2014). Correspondingly, in a constant‐temperature experiment, oligomers were generated at higher temperatures in the presence of the compound than in its absence when visualised by non‐denaturing PAGE (Fig 4B). Native state stability can also be probed by equilibrium unfolding using chemical denaturants, where a peak in bis‐ANS fluorescence corresponds with a maximally populated unfolding intermediate (James & Bottomley, 1998). The profiles in Fig 4C show that for guanidinium hydrochloride‐induced unfolding, this point occurs at a considerably higher denaturant concentration (~ 1.9 M) in the presence of 50 µM GSK716, than in its absence (~ 1.3 M), reflecting an increase in the stability of the native‐like state with respect to an unfolding intermediate. The association of GSK716 induced a marked quenching and blue‐shift of the α_1_‐antitrypsin intrinsic tryptophan fluorescence spectrum (Fig 4D, inset). The rate of change in fluorescence during association of 10µM GSK716 was proportional to the propensity of α_1_‐antitrypsin mutants to form polymers *in vivo*: inert (M), mild (S), moderate (Baghdad) and severe (Z) α_1_‐antitrypsin (Fig 4D, top and bottom).

To investigate whether this activity was consistent with action as a chemical chaperone, M α_1_‐antitrypsin and Z α_1_‐antitrypsin were unfolded *in vitro* into 6 M guanidine hydrochloride and rapidly refolded by snap dilution into denaturant‐free buffer in the presence or absence of GSK716. Electrophoresis of the products by non‐denaturing PAGE showed an anodally shifted migration for the Z variant in the absence of compound (Fig 4E) consistent with a misfolded by‐product of M* (Ekeowa *et al*, 2010), which was corrected at stoichiometric concentrations and above.

**Figure 5:**
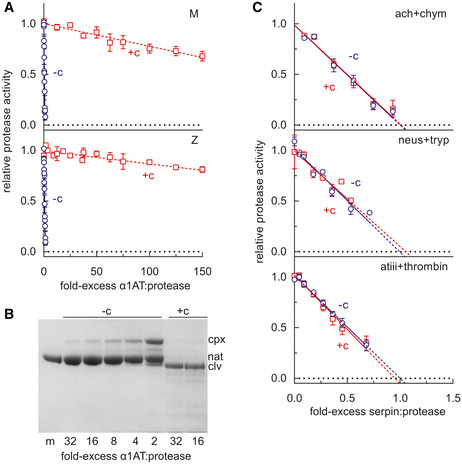
The effect of GSK716 on serpin inhibitory activity α_1_‐Antitrypsin was incubated at varying molar ratios with the model protease bovine α‐chymotrypsin, and the residual protease activity determined. The intercept of the regression with the abscissa reflects the number of molecules of α_1_‐antitrypsin required to inhibit one molecule of chymotrypsin in the presence (+c) and absence (−c) of 50µM GSK716. Error bars reflect ± SEM of three independent experiments.M α_1_‐antitrypsin was incubated with different molar ratios of chymotrypsin and resolved by SDS–PAGE. The position of covalent α_1_‐antitrypsin‐chymotrypsin complex (cpx), native (nat) and cleaved (clv) is shown.The inhibitory activity of α_1_‐antichymotrypsin against chymotrypsin (ach + chym), neuroserpin against trypsin (neus + tryp), and antithrombin against thrombin (atiii + thrombin) was determined in the presence and absence of GSK716. Error bars reflect ± SD of two independent experiments.

Mutations that interfere with the opening of β‐sheet A or that perturb its interaction with the N‐terminal portion of the reactive centre loop alter the ability of serpins to inhibit target proteases (Hood *et al*, 1994; Irving *et al*, 2014). The stoichiometry of inhibition (SI) was determined for M α_1_‐antitrypsin and Z α_1_‐antitrypsin in discontinuous experiments against a model target protease, chymotrypsin. The pre‐incubation of both variants with GSK716 led to a > 98% loss of protease inhibitory activity (Fig 5A). Resolution of the products of the interaction by SDS–PAGE showed full cleavage of the reactive centre loop (Fig 5B); therefore, this is not a consequence of the protease recognition site in the reactive centre loop becoming inaccessible to the enzyme. These data are consistent with a mechanism in which the compound stabilises β‐sheet A against conformational change that mediates both inhibitory activity and pathological misfolding.

### Characterisation of drug‐like properties of GSK716

GSK716 selectivity and PK properties were profiled in order to investigate the suitability of GSK716 for progression into *in vivo* studies and the potential for taking it forward as a clinical candidate for testing in humans. Since GSK716 results in loss of inhibitory activity of α_1_‐antitrypsin, the effect of the compound was assessed on other closely related serpins. GSK716 did not affect the inhibitory activity of antithrombin, neuroserpin and α_1_‐antichymotrypsin towards their cognate proteases (Fig 5C). Furthermore, there were no off‐target effects in a panel of assays considered predictive of known safety liabilities that precluded further development of GSK716 (Table EV1).

Since GSK716 exhibited a good level of selectivity over the off‐target panel and over other serpins, we determined the *in vitro* and *in vivo* PK properties of the molecule with a view to exploring target engagement *in vivo*. GSK716 has a measured ChromLogD (pH7.4) of 3.8, low binding to human serum albumin (84.2%) and good solubility of amorphous drug substance in FaSSIF (969 μg/ml). Permeability in MDR1‐MDCK cells in the presence of pgp inhibitor GF120918 was high at 248 and 240 nm/s for the apical to basal and basal to apical directions, respectively (N53531‐23 for DI). GSK716 exhibited low metabolic clearance in human hepatocytes (0.31 ml/min/g tissue), with moderate to high clearance in mouse hepatocytes (4.56 ml/min/g tissue). It exhibited weak time‐dependent inhibition of CYP3A4 resulting in a 1.59‐fold shift in IC50. Taken together, oral bioavailability is predicted to be high in humans, with measured F in rat (48%) and dog (71%) at ≤ 3 mg/kg being consistent with 100% absorption and losses via first‐pass metabolism only. Mean exposure of GSK716 in blood in the male CD‐1 mouse increased with dose following single PO administration at 10, 30 or 100 mg/kg (mean dose‐normalised *C*_max_ 58 ± 112, 113 ± 27 and 113 ± 27; DNAUC_inf_ 202 ± 101, 294 ± 47 and 403 ± 246, respectively).

### GSK716 increases secretion of Z α_1_‐antitrypsin in a transgenic mouse model of Z α_1_‐antitrypsin deficiency

GSK716 was evaluated in a transgenic mouse model with an engineered random insertion of the human Z α_1_‐antitrypsin gene (Teckman *et al*, 2004). Younger hemizygous, rather than older mice, were selected as our longevity studies showed that circulating levels of Z α_1_‐antitrypsin increase with age (to levels much higher than seen in patients) and these artificially high levels may act as a high‐affinity sink sequestering drug and preventing bioavailability at the site of action in the liver. Further, older mice have larger α_1_‐antitrypsin inclusions that may be more difficult to reverse and any changes would be more difficult to detect than in younger animals. The PK‐PD relationship of GSK716 was explored by dosing Z α_1_‐antitrypsin transgenic animals with 10, 30 or 100 mg/kg GSK716 three times a day. Blood and liver were harvested on day 6 at 3 h (~ *C*_max_) and 8 h (*C*_min_) after the dose for the measurement of total and free drug in both tissues. Blood was also harvested for the measurement of monomeric Z α_1_‐antitrypsin in plasma. Total concentrations of GSK716 were determined by LC‐MS/MS, and the free drug in both tissues was determined using equilibrium dialysis to determine free fraction in the samples, subsequently used to derive unbound concentrations. Blood concentrations demonstrated that the *C*_min_ levels of free drug were at or above 300 nM, the cellular secretion assay EC50, for the majority of the dosing period following 100 mg/kg dosing, whereas 30 and 10 mg/kg doses resulted in free drug levels in blood significantly below the cellular EC50 concentrations for a large part of the dosing period. Both free and total drug concentrations of GSK716 at the targeted site of action in the liver were equivalent to those in blood (Table 2).

**Table 2:** Unbound and total drug concentrations of GSK716 following dosing in Z α_1_‐antitrypsin transgenic mice.

Time (h)	GSK716 10 mg/kg TOTAL		GSK716 30 mg/kg TOTAL		GSK716 100 mg/kg TOTAL		GSK716 10 mg/kg UNBOUND		GSK716 30 mg/kg UNBOUND		GSK716 100 mg/kg UNBOUND	
	Mean concentration ± SD (µM)											
	10 mg/kg Blood	10 mg/kg Liver	30 mg/kg Blood	30 mg/kg Liver	100 mg/kg Blood	100 mg/kg Liver	10 mg/kg Blood	10 mg/kg Liver	30 mg/kg Blood	30 mg/kg Liver	100 mg/kg Blood	100 mg/kg Liver
123	8.7 (± 0.3)	3.5 (± 2.1)	14 (± 1.1)	6.7 (± 1.6)	33 (± 2)	40.9 (± 19.1)	0.018 (± 0.006)	0.035 (± 0.01)	0.061 (± 0.027)	0.091 (± 0.05)	0.49 (± 0.151)	1.046 (± 0.3)
128	7.7 (± 1.4)	2.1 (± 0.36)	13 (± 1.6)	8.3 (± 3.3)	20 (± 2.5)	28.4 (± 23.5)	0.009 (± 0.006)	0.028 (± 0.01)	0.051 (± 0.024)	0.075 (± 0.04	0.18 (± 0.141)	0.679 (± 0.7)
339					30 (± 5.1)	84.6 (± 24.9)					0.529 (± 0.204)	
483					22 (± 4.4)	42.4 (± 17.5)					0.561 (± 0.227)	

**Figure 6:**
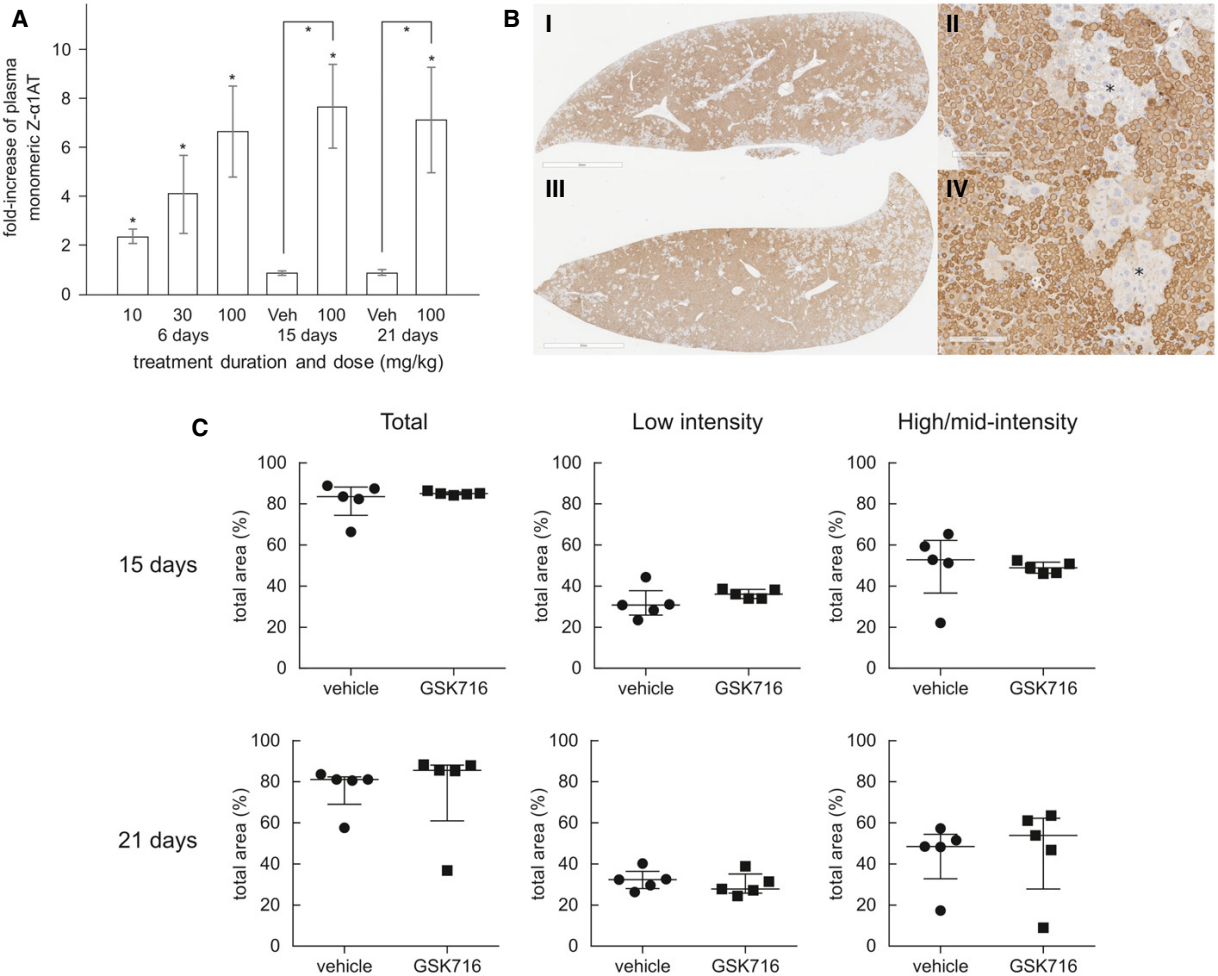
GSK716 increases secretion of Z α_1_‐antitrypsin in a transgenic mouse model of Z α_1_‐antitrypsin deficiency Z α_1_‐antitrypsin transgenic animals were dosed with 10, 30 or 100 mg/kg GSK716 three times a day. Total concentrations of GSK716 were determined by LC‐MS/MS (Table 2) 100 mg/kg GSK716 resulted in a mean sevenfold increase in circulating monomeric Z α_1_‐antitrypsin levels demonstrating robust target engagement in the liver. The observed steady‐state total and free compound levels of GSK716 in the transgenic Z α_1_‐antitrypsin mouse were well‐predicted by the *in silico* PK model built on: (i) *in vitro* metabolic clearance data, (ii) plasma protein binding data, (iii) *in vivo* PK data from wild‐type mice and (iv) a term comprising a 5 μM circulating sink for drug with an affinity of 1.5 nM, representing the Z α_1_‐antitrypsin within blood. The target‐free drug concentration was selected based on the observed potency in the *in vitro* secretion assays in which the total drug approximates to the free drug in the assay. Interestingly, 30 mg/kg and 10 mg/kg groups also gave significant, dose‐dependent increases in circulating Z α_1_‐antitrypsin despite free concentrations being below the cellular EC50 for secretion for much or all of the dosing period. Significance at *P* < 0.05 by a Student *t*‐test is denoted by *, shown as pairwise comparisons (each animal is compared with itself, treated vs pre‐treatment) and between groups (compound treated vs vehicle), with *n* = 7, 8 and 8 per group for day 6 dose level 10, 30 and 100 mg/Kg, respectively; day 15, *n* = 10; day 21, *n* = 5. Data presented as mean ± SD.Representative example images of 2C1‐stained livers from a 20‐day vehicle (i and ii) and 100 mg/kg TID GSK716 (iii and iv)‐treated animals. The asterisks indicate regions of hepatocytes that are negative on 2C1 immunostaining. Scale bar (i) and (iii) 2mm and (ii) and (iv) 100 µm.Quantification of 2C1 stained area in livers from vehicle and 100 mg/kg TID GSK716‐treated animals at day 15 (top) and 21 (bottom), shown as total area and low‐only or high and mid‐only intensity‐stained areas. There were no significant differences between groups by Mann–Whitney *U*‐test; graphs show median ± interquartile ranges, with *n* = 5 per group.

A significant fraction of the total Z α_1_‐antitrypsin in the circulation is in the polymeric conformation (Tan *et al*, 2014). There are no antibodies that are specific for monomeric Z α_1_‐antitrypsin and so to directly determine its concentration, a deconvolution method was developed based on immunoassays with antibodies for either total or polymeric α_1_‐antitrypsin, and calibration curves with purified monomeric and polymeric Z α_1_‐antitrypsin. Monomeric Z α_1_‐antitrypsin was measured in plasma samples following 6 days of dosing, and levels were normalised to each animals’ pre‐dose control levels to account for the natural variation of Z α_1_‐antitrypsin between animals. Administration of 100 mg/kg GSK716 resulted in a mean sevenfold increase in circulating monomeric Z α_1_‐antitrypsin levels demonstrating robust target engagement in the liver (Fig 6A). Interestingly, 30 and 10 mg/kg groups also gave significant, dose‐dependent increases in circulating Z α_1_‐antitrypsin despite free concentrations being below the cellular EC50 for secretion for much or all of the dosing period. Total drug levels and changes in Z α_1_‐antitrypsin following 3 days of dosing were indistinguishable from those following 6 days of dosing. There was no effect on circulating serum albumin after 5 days of dosing which provides evidence that GSK716 is specific for Z α_1_‐antitrypsin. Moreover, the effects are not mediated by metabolites of GSK716 as the major metabolites have much reduced or no binding to α_1_‐antitrypsin.

**Figure EV1:**
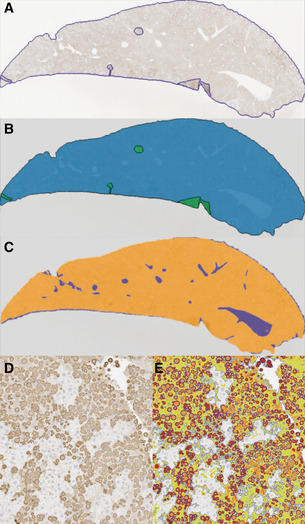
Summary of image analysis Liver section stained with 2C1 IHC with perimeter and artefacts drawn around freehand.Mask of (A), green areas are excluded from quantification.Mask of (A) with white space (blue areas) removed from quantification.Liver section stained with 2C1 IHC.Section in (D) marked up using thresholds set by a Board‐Certified pathologist. High intensity (red) and medium intensity (orange) areas describe the larger cytoplasmic inclusions, and low intensity (green) areas describe more diffuse cytoplasmic staining (smaller polymers). Algorithm created using Definiens Tissue Studio version 2.7 software whilst blind to sample identity.

Since GSK716 blocks polymer formation in cells, we explored the effect of dosing GSK716 on liver polymer levels. Z α_1_‐antitrypsin polymers formed in CHO‐TET‐ON‐Z‐A1AT and ZZ‐iPSC hepatocytes are cleared from cells with a half‐life of between 8 and 48 h depending on whether they partition to the soluble or insoluble fractions (Ronzoni *et al*, 2020). Since the compound does not bind Z α_1_‐antitrypsin polymer, an effect on total liver polymer levels will be dependent on the rate at which the liver can clear the polymer already present and the rate at which polymer continues to accumulate in animals not treated with drug. GSK716 was dosed at 100 mg/kg three times a day for 20 days. Monomeric Z α_1_‐antitrypsin increased by a mean of sevenfold to eightfold in plasma samples over the pre‐dosing baseline levels on days 15 and 21 of dosing, similar to the effect in animals dosed for 3 or 6 days and consistent with sustained target engagement through the dosing period (Fig 6A). Liver polymer levels were investigated by staining with 2C1 anti‐polymer monoclonal antibody and were scored blind by a pathologist or by quantification using an algorithm to measure all areas of positive staining (Fig EV1). There was no difference observed in total liver polymer load when assessed by manual or quantitative scoring (Fig 6B and C). There was no significant fibrosis in any of the liver sections. Intrahepatic α_1_‐antitrypsin was also assessed by ELISA. The vast majority (95–100%) of α_1_‐antitrypsin within the liver was polymer with the monomer typically being below the level of detection. Treatment with GSK716 increased the monomer measured in liver homogenate by approx. fourfold in keeping with the changes seen in blood.

## Discussion

Disrupting protein–protein interactions with small molecule compounds that maintain drug‐like properties is a significant challenge. Here, we describe the identification of a potent and selective α_1_‐antitrypsin corrector, GSK716 that abolishes intracellular polymerisation of Z α_1_‐antitrypsin and increases the circulating levels of monomeric protein by sevenfold in a transgenic mouse model of disease. The co‐crystal structure with α_1_‐antitrypsin demonstrates that the small molecule ameliorates the effect of the Glu342Lys (Z) mutation by: (i) optimisation of hydrophobic packing in the breach region; (ii) formation of hydrogen bonds with buried polar atoms; and (iii) displacement of the backbone at the top of strand 5A into a configuration less compatible with partial loop insertion. This movement of strand 5A is an early step in reactive loop‐β‐sheet A models of polymerisation (Gooptu *et al*, 2000) and an obligate one in the C‐terminal polymer linkage (Yamasaki *et al*, 2011). Following GSK716 binding, there is a marked stabilisation of α_1_‐antitrypsin against the conformational changes associated with M* intermediate formation. This in turn increases folding efficiency, thereby reducing the formation of polymers. Precedent for this general mechanism comes from our development of a tool monoclonal antibody that exerted a similar effect on Z α_1_‐antitrypsin (Ordóñez *et al*, 2015; Motamedi‐Shad *et al*, 2016).

The GSK716‐associated displacement at the top of strand 5A is consistent with the association rate‐driven preference for Z α_1_‐antitrypsin and an increased availability of the cryptic pocket. The pocket, once formed, appears to be structurally equivalent in both wild‐type M and mutant Z α_1_‐antitrypsin, as reflected by a similar rate of dissociation of GSK716. Binding of GSK716 to α_1_‐antitrypsin increased with the propensity of mutants to form polymers (M < S < Baghdad < Z α_1_‐antitrypsin), indicating that pocket formation and polymerisation are intimately linked. GSK716 stabilises the partially folded α_1_‐antitrypsin or the fully folded but labile aberrant native form. This mode of action is compatible with the lack of binding of GSK716 to polymers, in which partial or complete insertion of the reactive centre loop completes a β‐hairpin turn and so occludes the compound binding site.

GSK716 blocks Z α_1_‐antitrypsin polymerisation in cell‐free media and in the ER of both CHO and iPSC models of α_1_‐antitrypsin deficiency. It increased secretion from the iPSC model by approximately threefold. Treatment with GSK716 reduced the levels of intracellular Z α_1_‐antitrypsin polymer compared with cells assessed before compound addition demonstrating that polymers can be cleared over the time course of the experiment and that accumulation of polymers is reversible in ZZ‐iPSC‐hepatocytes. These findings were confirmed by pulse‐chase experiments which showed that GSK716 abolished intracellular polymers (when assessed by the 2C1 mAb) and increased the clearance and secretion of Z α_1_‐antitrypsin. Dosing of GSK716 in transgenic mice that express Z α_1_‐antitrypsin increased circulating levels of Z α_1_‐antitrypsin by sevenfold within 3 days, indicating robust target engagement. This effect was maintained to the conclusion of the experiment at day 21. The increase in Z α_1_‐antitrypsin in the circulation at 10 and 30 mg/kg of GSK716 was surprising given that systemic free drug levels were below the cellular EC50 for secretion for much or all of the dosing period. The reason for this is unclear but it is possible that the target engagement *in vivo* is greater than predicted from the potency in the *in vitro* cellular assays. Alternatively, it is possible that the first‐pass effect of drug reaching the liver immediately after absorption delivers some efficacy over that predicted from modelling the compound concentration at steady‐state levels. Together, these data suggest potential upsides for the required compound exposure to deliver efficacy in individuals with Z α_1_‐antitrypsin deficiency.

Despite the increase in plasma levels of Z α_1_‐antitrypsin, there was no reduction in intrahepatic Z α_1_‐antitrypsin inclusions after 20 days of dosing in the transgenic mouse. This may be because the Z α_1_‐antitrypsin is released from the globule‐devoid hepatocytes or because the intrahepatic polymers in the transgenic mice are not cleared as readily as those that are generated over a few days in model systems such as CHO cells and iPSC hepatocytes. Our findings are in contrast to work with the autophagy activator carbamazepine which had profound effects on liver polymer in Z α_1_‐antitrypsin transgenic mice following 2 weeks of dosing (Hidvegi *et al*, 2010). RNAi approaches that inhibit Z α_1_‐antitrypsin expression and polymer formation have reported decreases in Z α_1_‐antitrypsin in transgenic mouse liver following 12–33 weeks of treatment, albeit without reports of data at earlier timepoints (Guo *et al*, 2014). It is likely that GSK716 will need to be dosed to transgenic Z α_1_‐antitrypsin mice for significantly longer than 20 days to demonstrate an effect on total liver polymer levels. It remains to be seen whether the intrahepatic polymer needs to be cleared from the liver in order to have some functional benefit or whether the accumulated polymer inclusions are inert and abrogation of polymer production is sufficient to restore the functioning of the ER and hence the health of the cells. Moreover, treatment with GSK716 may be sufficient to protect against the two‐hit process whereby the Z α_1_‐antitrypsin polymers sensitise the liver to a secondary insult such as alcohol, drug or liver fat (Ordóñez *et al*, 2013; Strnad *et al*, 2019).

Polymer formation and inhibitory activity are inextricably linked in the serpin mechanism (Gooptu & Lomas, 2009), and thus, small molecules that block polymerisation may have the unwanted effect of also blocking inhibitory activity. Bound GSK716 inhibits the serpin activity of α_1_‐antitrypsin and so would not be expected to increase protease inhibitory activity during the dosing period. However, the half‐life of monomeric Z‐α_1_‐antitrypsin in humans is ~ 6 days whereas the drug would be expected to be cleared with a half‐life of a few hours after dosing, raising the possibility of a pulsatile dosing regimen that would lead to increased, active serpin. The slow development of the lung disease in individuals with α_1_‐antitrypsin deficiency over many decades suggests that acute effects associated with inhibition of serpin activity are unlikely.

There is increasing recognition that heterozygosity for wild‐type M and mutant Z α_1_‐antitrypsin alleles predisposes to liver disease (Strnad *et al*, 2019). Our small molecule approach to block polymer formation has an advantage over siRNA therapies to “knockdown” α_1_‐antitrypsin production in treating the MZ α_1_‐antitrypsin heterozygote. GSK716 has a 100‐fold greater affinity for Z than M α_1_‐antitrypsin and so may be dosed at a level that prevents polymerisation of Z α_1_‐antitrypsin without reducing the inhibitory effect of the wild‐type M protein.

In summary, we report the first small molecule drug‐like correctors of Z α_1_‐antitrypsin folding obtained via optimisation of hits from an encoded library technology screen (Clark *et al*, 2009; Arico‐Muendel, 2016; Goodnow *et al*, 2017) that are suitable for oral delivery, correct folding in human patient iPSC‐derived hepatocytes and increase circulating Z α_1_‐antitrypsin levels in a transgenic mouse model of α_1_‐antitrypsin deficiency.

## Materials and Methods

Alpha_1_‐antitrypsin was purified from the plasma of M (wild‐type) and Z α_1_‐antitrypsin homozygotes, and recombinant Cys232Ser α_1_‐antitrypsin was expressed and purified as detailed previously (Lomas *et al*, 1993; Irving *et al*, 2011; Haq *et al*, 2013). The data generated in this manuscript have used a number of different preparations of GSK716 all of which have been checked for identity and purity by NMR/MS. Moreover, all batches have been tested for activity in blocking α_1_‐antitrypsin polymerisation in the assay as shown in Fig 1B. Full experimental procedures and analytical data for synthesising GSK716 are available in patent WO2019/243841A1 (“compound 1”). The experiments conformed to the principles set out in the WMA Declaration of Helsinki and the Department of Health and Human Services Belmont Report. All animal studies were ethically reviewed and carried out in accordance with European Directive 2010/63/EEC and the GSK Policy on the Care, Welfare and Treatment of Animals, or by the ethical review process at the institution where the work was performed.

### DNA‐encoded library technology screen

An encoded library technology (ELT) screen with a nominal diversity of 2 × 10^12^ unique components was used to identify small molecules that bind monomeric Z α_1_‐antitrypsin at 4, 22 and 37°C. Affinity selections were performed as described previously (Clark *et al*, 2009).

### *In vitro* assay of Z α_1_‐antitrypsin polymerisation

An antibody‐based time‐resolved fluorescence resonance energy transfer (TR‐FRET) assay was developed to monitor the polymerisation of 5 nM Z α_1_‐antitrypsin following incubation with varying concentrations of compounds at 37°C for 72 h. This assay used the 2C1 monoclonal antibody (1.25 nM) that is specific to pathological polymers of α_1_‐antitrypsin (Miranda *et al*, 2010), a polyclonal antibody (1/320 dilution) that binds to all forms of α_1_‐antitrypsin (Abcam product 9373), an anti‐mouse IgG (1.5 nM) labelled with fluorescence donor Eu‐W1024 (Perkin Elmer product AD0076) and an anti‐rabbit IgG (14.3 nM labelled with acceptor [APC]). In the presence of polymeric Z α_1_‐antitrypsin, a 4‐antibody sandwich is formed allowing energy transfer to occur between the Europium‐ and Allophycocyanin fluorophores. The TR‐FRET signal was read on an Envision plate reader (PerkinElmer), by excitation of Europium at 337 nm and detection of emission at 665 and 620 nm.

### Compound association experiments

Kinetic parameters of GSK716 binding to M, Z, S (Glu264Val) and Baghdad (Ala336Pro) (Haq *et al*, 2016) α_1_‐antitrypsin were measured by detecting intrinsic tryptophan fluorescence of the protein (excitation at 280 nm and detection of emission at 320 nm) on a stopped flow apparatus (Applied Photophysics) (Kim & Yu, 1996; Dafforn *et al*, 1999). A competition assay for binding to M α_1_‐antitrypsin and Z α_1_‐antitrypsin and Z α_1_‐antitrypsin polymers (Lomas *et al*, 1993; Irving *et al*, 2011; Haq *et al*, 2013), based on an Alexa488‐labelled analogue of GSK716 (A488‐GSK716), was used to determine the binding affinity of test compounds.

### Thermal stability, unfolding and assessment of protease inhibition

The native state stability of α_1_‐antitrypsin on addition of compounds was investigated by thermal denaturation in the presence of a 5X concentration of SYPRO Orange dye solution (Life Technologies) (Nettleship *et al*, 2008). Resistance to heat‐induced polymerisation was determined using an end‐point constant‐temperature assay. Equilibrium unfolding was evaluated with a bis‐ANS dye (Dafforn *et al*, 1999), and rapid refolding following denaturation in 6 M urea was assessed by non‐denaturing PAGE. The inhibitory activity of α_1_‐antitrypsin was measured by titration against the model protease bovine α‐chymotrypsin. The activity of antithrombin, neuroserpin and α_1_‐antichymotrypsin was assessed against human thrombin, bovine trypsin and bovine α‐chymotrypsin, respectively.

### Crystallisation and structure determination

Crystallisation of recombinant Cys232Ser α_1_‐antitrypsin was carried out in 2‐well MRC crystallisation plates with a Mosquito robot (TTP Labtech) using 100 nl protein solution and 100 nl well solution. Crystals grew from 22% w/v PEG1500, 0.2 M MES pH6.0 and were soaked for 24 h with 25 mM compound (5% v/v DMSO). X‐ray diffraction data were collected at Diamond on beamline I03. Structure solution was carried out by molecular replacement using PHASER (McCoy *et al*, 2007). The model used to solve this structure was a related complex (data not shown) which had been solved by molecular replacement using PDB entry 2QUG as a starting model. Building was carried out using Coot (Emsley *et al*, 2010) and refinement with REFMAC (Murshudov *et al*, 1997).

### *In vitro* pharmacokinetics

ChromLogD was measured as previously described (Valkó *et al*, 1997).

#### Permeability in MDR1‐MDCK cells with pgp inhibitor

MDR1‐MDCK cells were used between passage numbers 6–30. Cells were seeded onto Millipore Multiscreen Transwell plates at 3.4 × 105 cells/cm^2^. The cells were cultured in DMEM and media was changed on day 3. On day 4 the permeability study was performed. Cell culture and assay incubations were carried out at 37°C in an atmosphere of 5% *v*/*v* CO_2_ with a relative humidity of 95%. On the day of the assay, the monolayers were prepared by rinsing both apical and basolateral surfaces twice with Hanks Balanced Salt Solution (HBSS) at the desired pH warmed to 37°C. Cells were then incubated with HBSS at the desired pH in both apical and basolateral compartments for 40 min to stabilise physiological parameters. Where applicable, a P‐gp inhibitor (elacridar, 2 µM) was included on both sides of the monolayer for the equilibration period. For assessment of apical‐basolateral permeability, HBSS was removed from the apical compartment and replaced with test compound dosing solution. The apical compartment insert was then placed into a companion plate containing fresh buffer (containing 0.5% *v*/*v* DMSO or, where applicable, a P‐gp inhibitor, maintaining a 0.5% *v*/*v* DMSO concentration). For assessment of basolateral‐apical permeability, HBSS was removed from the companion plate and replaced with test compound dosing solution. Fresh buffer (containing 0.5% *v*/*v* DMSO or, where applicable, a P‐gp inhibitor, maintaining a 0.5% *v*/*v* DMSO concentration) was added to the apical compartment insert, which was then placed into the companion plate. At 60 min the apical compartment inserts and the companion plates were separated and apical and basolateral samples diluted for analysis.

#### CYP3A4 TDI fold IC50 shift

Seven concentrations of GSK716 (2, 4, 10, 20, 40, 100 and 200 µM) plus a vehicle control (0.25% *v*/*v* DMSO in pre‐incubation) were pre‐incubated at 37°C with human liver microsomes (0.1 mg/ml) and NADPH (1 mM) for a range of five pre‐incubation times (5, 10, 15, 20 and 30 min) with a 0 min pre‐incubation, in duplicate. At the end of the individual pre‐incubations, an aliquot of the pre‐incubation mixture was added to an incubation mixture in a 1:20 dilution for GSK716 with the specific CYP3A4 probe substrate, midazolam (12.5 µM, equivalent to 5xKm44) and NADPH (1 mM) for a 5 min. The reactions were terminated by transferring an aliquot of incubation mixture to methanol. The samples were mixed and then centrifuged at 2,500 rpm for 30 min at 4°C. Aliquots of the supernatant were diluted with formic acid (final concentration 0.1 % *v*/*v*) in deionised water containing internal standard (metoprolol; final concentration 0.03 mg/l). Samples were analysed by LC‐MS/MS.

#### Hepatocyte clearance

Williams E media supplemented with 2 mM l‐glutamine and 25 mM HEPES and test compound (final compound concentration 0.5 µM; final DMSO concentration 0.25% *v*/*v*) were preincubated at 37°C prior to the addition of a suspension of cryopreserved pooled hepatocytes (final cell density 0.5 × 106 viable cells/ml) to initiate the reaction. The final incubation volume was 500 µl. The reactions were stopped by transferring 50 µl of incubate to 100 µl acetonitrile containing internal standard at the appropriate time points (0, 5, 10, 20, 40 and 60 min, 0, 10, 20, 40, 60 and 120 min or 0, 20, 40, 60, 120 and 240 min). The control (lysed cells or vehicle) was incubated for 60, 120 or 240 min only. The termination plates were centrifuged at 2,500 rpm at 4°C for 30 min to precipitate the protein. Following protein precipitation, the sample supernatants were analysed by LC‐MS/MS. From a plot of loge peak area ratio (compound peak area/internal standard peak area) against time, the gradient of the line was determined and half‐life (*t*½) and intrinsic clearance (CLint) were calculated.

### Measurement of monomeric Z α_1_‐antitrypsin in plasma and cell biology

Plasma standards of monomeric and polymeric Z α_1_‐antitrypsin were prepared and assayed with an antibody mix comprising a 1:160 dilution of rabbit polyclonal anti‐ α_1_‐antitrypsin (Abcam product 9373), 0.23 µg/ml terbium anti‐mouse, 4.5 µg/ml Alexa488 goat anti‐rabbit, 0.17 µg/ml mouse anti‐total‐ α_1_‐antitrypsin monoclonal 3C11 and 2 µg/ml mouse 2C1 anti‐polymeric‐ α_1_‐antitrypsin. After a 16‐h incubation at 20°C, TR‐FRET detection was performed on an Envision plate reader (Perkin Elmer).

Plots of the FRET ratio (acceptor/donor signal) versus sample dilution result in bell‐shaped curves due to the hook effect. The polymer Z α_1_‐antitrypsin concentration was determined by comparing the peak positions of these bell‐shaped curves for plasma samples with those for polymer calibration samples. The polymer concentration in the plasma sample and the polymer calibration curve was then used to derive the contribution of polymer to the FRET signal in the total α_1_‐antitrypsin assay at the signal peak, enabling determination of the concentration of monomeric Z α_1_‐antitrypsin.

#### Z α_1_‐antitrypsin secretion / polymerisation in CHO‐TET‐ON‐Z‐α_1_AT cells

The accumulation and clearance of Z α_1_‐antitrypsin polymer was measured with the 2C1 monoclonal antibody that is specific for polymerised antitrypsin in CHO‐TET‐ON‐Z‐α_1_AT cells (Ordóñez *et al*, 2013) and iPSC‐derived hepatocytes generated from a patient with a PiZZ genotype (Yusa *et al*, 2011). Secretion of Z α_1_‐antitrypsin was determined by TR‐FRET. Cell viability was analysed with Cell Counting Kit‐8 (Sigma) after treating the cells with 10 µM GSK716 or 0.1% v/v DMSO (vehicle) and an increasing concentration of tunicamycin (0.8–0.01 µg/ml).

#### Pulse chase experiments

CHO K1 cells were labelled after 48‐h induction with 0.5 μg/ml doxycycline. Cells were pulsed (0.45 MBq/10^6^ cells) for 10 min with ^35^S Cys/Met (EasyTagTM Express Protein Labelling, Perkin Elmer, Beaconsfield, UK) in DMEM without Cys/Met, and then chased in normal culture medium for 0, 1, 4, 8 and 12 h. Radiolabelled α_1_‐antitrypsin was isolated by immunoprecipitation and resolved by SDS–PAGE followed by autoradiography. Densitometric analysis of α_1_‐antitrypsin bands was performed with ImageStudioLite (LI‐COR Biosciences, USA). Statistical analysis was performed using the GraphPad Prism program (GraphPad Software, La Jolla, CA, USA).

### *In vivo* experiments

#### Pharmacokinetics

Male CD‐1 mice, Han Wistar rats or beagle dogs were administered GSK716 as a suspension in 1% w/v aqueous methylcellulose via oral gavage at doses of 1 mg/kg (dog) or 10, 30 and 100 mg/kg (mouse, rat). Blood samples were taken into EDTA, diluted and mixed with acetonitrile containing internal standard (alprazolam) and centrifuged to precipitate proteins. Aliquots of the resultant supernatant were analysed by LC‐MS/MS, and concentrations of GSK716 in blood were determined. Non‐compartmental pharmacokinetic analysis (NCA) was carried out using Phoenix WinNonLin 6.3 (Certara L.P.). Free drug was measured in *Z* α_1_‐antitrypsin transgenic mice by dialysing tissue samples in rapid equilibrium dialysis cassettes.

#### Z α_1_‐antitrypsin transgenic mouse experiments

Hemizygous female Z α_1_‐antitrypsin transgenic mice were 13–15 weeks of age at start of study. Drugs were administered via oral gavage three times a day at 8‐h intervals at 10 ml/Kg as a suspension in aqueous methylcellulose containing 0.5% w/v Tween 80. In life, blood sampling was performed vial tail nick. Animals were housed in licensed, approved housing in the UK with appropriate enrichment. Animals were randomly assigned to groups using manual methods. Histopathology analysis was performed blind.

#### Histopathology, immunohistochemistry and image analysis

Formalin‐fixed, paraffin‐embedded livers from all mice were sectioned and stained with haematoxylin and eosin (H&E) and periodic acid Schiff (PAS) with diastase to examine tissue quality and the presence of diastase‐resistant polymer inclusions, respectively. Immunohistochemistry for polymerised α_1_‐antitrypsin was performed using 2C1 monoclonal primary antibody and a Mouse‐on‐Mouse Polymer Kit (Abcam AB127055) for detection. Representative sections of liver from each mouse were blindly evaluated by a Board‐Certified Veterinary Pathologist and ranked in order of polymer content. A semi‐automated image analysis solution was developed to quantify total polymer content (Fig EV1). Thresholds were optimised to separate staining of the polymer inclusions (high and medium intensity) and more diffuse cytoplasmic staining (low intensity).

### Statistics

Formal power calculations were not performed for *in vivo* studies but *n* numbers were determined based on prior experience with the model and readout. Animals were randomly assigned to groups, using manual methods not randomisation tools. *In vivo* work was performed by individuals unfamiliar with the project, and *ex vivo* analysis was performed by different scientists to those executing the in life phase. Histopathology analysis was performed blinded.

## Author contributions

DAL, AB and ACP designed the programme of work. DAL, ACP, JAI, IU and DSH served on the joint UCL‐GSK DPAc project oversight board. JAI, IH, AJ, ADo, DK, JPH, MR, JR and MB undertook the biochemical assessment of the compounds; AOr, SJM, AD, MR, TJ and RR assessed the cell biology; CC, KJS and MN undertook structural biology studies; CA‐M, SB, JM, AOl, ZZ, SS and KL undertook encoded library technology screening; HD, PE, EJ, RT, LT and SW undertook PK, *in vivo* profiling and pathology and; AB, ADe, ND, DSH and JL undertook medicinal chemistry. All authors reviewed, revised and approved the final manuscript.

## Conflict of interest

Kate Smith, Alexis Denis, Nerina Dodic, John Liddle and David Lomas are inventors on patent PCT/GB2019/051761. The intellectual property has been transferred from GlaxoSmithKline to UCL Business who have licensed it to a third party.

## For more information

https://www.omim.org/entry/613490

https://www.alpha1.org, https://www.alpha1.uk

## Supporting information



Expanded View Figures PDF

Table EV1

Review Process File

## Data Availability

The data set produced in this study: alpha 1‐antitrypsin (C232S) complexed with GSK716 is available in the PDB identifier 7AEL (http://www.rcsb.org/pdb/explore/explore.do?structureId=7AEL).
