# Supplementary material for: Development of a small molecule that corrects misfolding and increases secretion of Z α1‐antitrypsin
Source: EMBO Mol Med. 2021 Jan 29;13(3):e13167. doi: 10.15252/emmm.202013167 (PMC7933930; doi:10.15252/emmm.202013167)
Supplement: Supplementary file 1 — Expanded View Figures PDF [file EMMM-13-e13167-s001.pdf]

Expanded View Figures

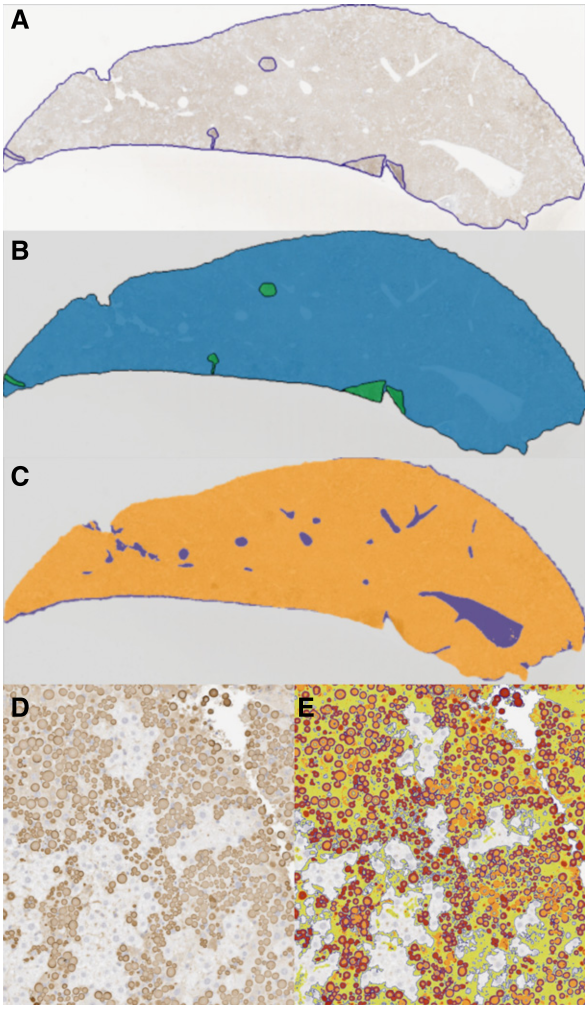

Figure EV1. Summary of image analysis.

A Liver section stained with 2C1 IHC with perimeter and artefacts drawn around freehand.
B Mask of (A), green areas are excluded from quantification.
C Mask of (A) with white space (blue areas) removed from quantification.
D Liver section stained with 2C1 IHC.
E Section in (D) marked up using thresholds set by a Board-Certified pathologist. High intensity (red) and medium intensity (orange) areas describe the larger cytoplasmic inclusions, and low intensity (green) areas describe more diffuse cytoplasmic staining (smaller polymers). Algorithm created using Definiens Tissue Studio version 2.7 software whilst blind to sample identity.
