# Supplementary material for: Development of a small molecule that corrects misfolding and increases secretion of Z α1‐antitrypsin
Source: EMBO Mol Med. 2021 Jan 29;13(3):e13167. doi: 10.15252/emmm.202013167 (PMC7933930; doi:10.15252/emmm.202013167)
Supplement: Supplementary file 2 — Table EV1 [file EMMM-13-e13167-s002.docx]

**Table EV1.**

|  |  |  |  | **#assay replicates** | |
| --- | --- | --- | --- | --- | --- |
| **Assay name** | **Mode** | **Mean (PXC50)** | **SD** | **Active** | **Inactive** |
| α1 nicotinic AChR | Blocker | 4.5 | 0.14 | 3 | 1 |
| α1 nicotinic AChR | Opener | <4.3 |  |  | 8 |
| Acetyl- cholinesterase | Inhibition | 4.0 |  | 1 | 9 |
| Adenosine A2a | Agonist | <4 |  |  | 1 |
| Adrenergic α1B | Antagonist | <4.6 |  |  | 8 |
| Adrenergic α1B (DRX) | Antagonist | <4.6 |  |  | 6 |
| Adrenergic α2C | Agonist | <4 |  |  | 2 |
| Adrenergic α2C (DRX) | Agonist | <4 |  |  | 6 |
| Adrenergic β2 | Agonist | <4 |  |  | 2 |
| Adrenergic β2 | Antagonist | <4 |  |  | 2 |
| Adrenergic β2 (DRX) | Agonist | <4 |  |  | 6 |
| Adrenergic β2 (DRX) | Antagonist | <4 |  |  | 6 |
| Aryl hydrocarbon receptor | Activator | <4 |  |  | 1 |
| Aurora B (STK12) | Inhibition | <4.5 |  |  | 1 |
| Bile Salt Export Pump | Inhibition | 4 | 0.7 | 4 | 6 |
| Cannabinoid CB1 (DRX) | Agonist | <4 |  |  | 4 |
| Cannabinoid CB2 | Agonist | <4 |  |  | 8 |
| Cell Health | Membrane Permeability | <3.7 |  |  | 11 |
| Cell Health | Mitochondrial Potential | 3.8 | 0.3 | 6 | 5 |
| Cell Health | Nucleus (size) | <3.7 |  |  | 11 |
| Cycloxygenase2 (COX2) | Inhibition | <4 |  |  | 1 |
| Dopamine D1 | Antagonist | <4 |  |  | 2 |
| Dopamine D1 (DRX) | Antagonist | <4 |  |  | 6 |
| Dopamine D2 | Agonist | <4 |  |  | 2 |
| Dopamine D2 | Antagonist | <4 |  |  | 2 |
| Dopamine D2 (DRD2S) (DRX) | Agonist | <4 |  |  | 6 |
| Dopamine D2 (DRD2S) (DRX) | Antagonist | <4 |  |  | 6 |
| GABA-A (Barracuda) | Agonist | <4 |  |  | 25 |
| GABA-A (Barracuda) | Antagonist | <4 |  |  | 16 |
| GABA-A (Barracuda) | Pos Mod | <4 |  |  | 13 |
| Glucocorticoid Receptor | Agonist | <4 |  |  | 1 |
| hERG (Barracuda) | Blocker | 4.8 |  | 1 | 19 |
| Histamine H1 | Antagonist | <4.6 |  |  | 8 |
| Histamine H1 (DRX) | Antagonist | 5 | 0.9 | 2 | 4 |
| KCNQ1/minK | Blocker | <4.6 |  |  | 12 |
| Kv1.5 | Blocker | <4.3 |  |  | 12 |
| L-type Ca channel (CaV1.2) | Blocker | >5.67 |  | 2 | 10 |
| LCK | Inhibition | <4.5 |  |  | 1 |
| Monoamine oxidase A | Inhibition | <4 |  |  | 1 |
| MRGPRX2 Agonist (DRX) | Agonist | <4 |  |  | 6 |
| Muscarinic M1 | Agonist | <4.3 |  |  | 16 |
| Muscarinic M1 | Antagonist | <4.3 |  |  | 16 |
| Muscarinic M1 (DRX) | Agonist | <4.3 |  |  | 6 |
| Muscarinic M1 (DRX) | Antagonist | <4.3 |  |  | 6 |
| Muscarinic M2 | Agonist | <4.3 |  |  | 8 |
| Muscarinic M2 | Antagonist | <4.3 |  |  | 8 |
| Muscarinic M2 (DRX) | Agonist | 5.3 | 0.42 | 4 | 2 |
| Muscarinic M2 (DRX) | Antagonist | <4.3 |  |  | 6 |
| NaV1.5 (IonWorks) | Blocker | 4.1 | 0.14 | 3 | 11 |
| NK1 (TACR1) | Antagonist | <4.6 |  |  | 8 |
| NK1 (TACR1) (DRX) | Antagonist | <4.6 |  |  | 6 |
| NMDA (NR2B) | Blocker | <4.3 |  |  | 2 |
| Norepinephrine transporter | Antagonist | <4 |  |  | 2 |
| Norepinephrine transporter (DRX) | Blocker | <4 |  |  | 6 |
| NULL CHO-K1 Agonist (DRX) | Agonist | <4 |  |  | 6 |
| OATP1B1 | Inhibition | <4.3 |  |  | 1 |
| OPRK1 | Agonist | <4 |  |  | 2 |
| OPRK1 (DRX) | Agonist | 4.7 | 0.99 | 4 |  |
| OPRM1 | Agonist | <4 |  |  | 2 |
| OPRM1 (DRX) | Agonist | 5 | 0.31 | 3 |  |
| PDE3A | Inhibition | <4 |  |  | 1 |
| PDE4B | Inhibition | <4 |  |  | 12 |
| Phospholipidosis | Induction (pMEC) | pMEC=  5.8 | 0.42 | 2 | 22 |
| PI3Kg | Inhibition | <4.5 |  |  | 6 |
| PXR (human) | Activator | 4.7 | 0.02 | 5 | 8 |
| PXR (rat) | Activator | 4.9 |  | 1 | 1 |
| Serotonin 5HT1B | Agonist | <4 |  |  | 2 |
| Serotonin 5HT1B | Antagonist | <4 |  |  | 2 |
| Serotonin 5HT1B (DRX) | Agonist | <4 |  |  | 4 |
| Serotonin 5HT1B (DRX) | Antagonist | <4 |  |  | 4 |
| Serotonin 5HT2A | Agonist | <4.6 |  |  | 8 |
| Serotonin 5HT2A | Antagonist | <4.6 |  |  | 8 |
| Serotonin 5HT2A (DRX) | Agonist | <4 |  |  | 6 |
| Serotonin 5HT2A (DRX) | Antagonist | 4.2 |  | 1 | 5 |
| Serotonin 5HT2B (DRX) | Agonist | 4.4 |  | 1 | 5 |
| Serotonin 5HT2C | Agonist | <4.6 |  |  | 8 |
| Serotonin 5HT2C | Antagonist | <4.6 |  |  | 8 |
| Serotonin 5HT2C (DRX) | Agonist | <4 |  |  | 6 |
| Serotonin 5HT2C (DRX) | Antagonist | 4.3 | 0.2 | 2 | 4 |
| Serotonin 5HT3 | Blocker | 4.5 | 0.1 | 3 | 5 |
| Serotonin 5HT3 | Opener | <4.3 |  |  | 8 |
| Serotonin 5HT3 (DRX) | Blocker | 4.5 | 0.3 | 4 | 2 |
| Serotonin 5HT3 (DRX) | Opener | <4.3 |  |  | 6 |
| Serotonin transporter | Antagonist | <4 |  |  | 2 |
| Serotonin transporter (DRX) | Blocker | <4 |  |  | 6 |
| Vasopressin V1a | Antagonist | 4.4 | 0.09 | 3 | 4 |
| Vasopressin V1a (DRX) | Antagonist | <4.3 |  |  | 6 |
