# Supplementary material for: Development of a small molecule that corrects misfolding and increases secretion of Z α1‐antitrypsin
Source: EMBO Mol Med. 2021 Jan 29;13(3):e13167. doi: 10.15252/emmm.202013167 (PMC7933930; doi:10.15252/emmm.202013167)
Supplement: Supplementary file 3 — Review Process File [file EMMM-13-e13167-s003.pdf]

EMBO Molecular Medicine

Development of a small molecule that corrects misfolding and increases secretion of Z α1-antitrypsin

David Lomas, James Irving, Christopher Arico-Muendel, Svetlana Belyanskaya, Andy Brewster, Murray Brown, Chun-wa Chung, Hitesh Dave, Alexis Denis, Nerina Dodic, Anthony Dossang, Peter Eddershaw, Diana Klimaszewska, Imran Haq, Duncan Holmes, Jon Hutchinson, Alistair Jagger, Toral Jakhria, Emilie Jigorel, John Liddle, Ken Lind, Stefan Marciniak, Jeff Messer, Margaret Neu, Allison Olszewski, Adriana Ordoñez, Riccardo Ronzoni, James Rowedder, Martin Rüdiger, Steven Skinner, Kate Smith, Rebecca Terry, Lionel Trottet, Iain Uings, Steve Wilson, Zhengrong Zhu, and Andrew Pearce
DOI: 10.15252/emmm.202013167

Corresponding authors: David Lomas (d.lomas@ucl.ac.uk) , Andrew Pearce (andrew.c.pearce@gsk.com)

Review Timeline:

Submission Date:	23rd Jul 20
Editorial Decision:	7th Sep 20
Revision Received:	10th Nov 20
Editorial Decision:	1st Dec 20
Revision Received:	8th Dec 20
Accepted:	10th Dec 20

Editor: Zeljko Durdevic

Transaction Report:

(Note: With the exception of the correction of typographical or spelling errors that could be a source of ambiguity, letters and reports are not edited. Depending on transfer agreements, referee reports obtained elsewhere may or may not be included in this compilation. Referee reports are anonymous unless the Referee chooses to sign their reports.)

7th Sep 2020

Dear Prof. Lomas,

Thank you for the submission of your manuscript to EMBO Molecular Medicine. We have now heard back from the three referees who agreed to evaluate your manuscript. As you will see from the reports below, while referee #1 and #2 are overall supporting publication of your work, referee #3 highlights the interest of the study but also raises a number of concerns that should be addressed in a major revision of the current manuscript. After a cross-commenting exercise it became clear that a control experiment with M alpha1-antitrypsin-expressing cells treated with GSK716 should be included, while no further in vivo experiments are required. However, addressing the reviewers' concerns in full, experimentally or in writing, will be necessary for consideration of your manuscript in our journal. Particularly, you should provide a detailed response to the referee #3 criticism of the mouse model, and in vivo experiments and discuss the limitations of the study in that regard. Also, PDB validation reports should be made available as suggested by the referee #1.

Please be aware that the acceptance of the manuscript will entail a second round of review. Please note that EMBO Molecular Medicine encourages a single round of revision only and therefore, acceptance or rejection of the manuscript will depend on the completeness of your responses included in the next, final version of the manuscript. For this reason, and to save you from any frustrations in the end, I would strongly advise against returning an incomplete revision.

We would welcome the submission of a revised version within three months for further consideration. However, we realize that the current situation is exceptional on the account of the COVID-19/SARS-CoV-2 pandemic. Please let us know if you require longer to complete the revision.

I look forward to receiving your revised manuscript.

***** Reviewer's comments *****

Referee #1 (Remarks for Author):

This paper represents a triumphant progress, led by the first author over a period of nearly 30 years,

from the initial proposal of a conformational basis for a common inherited disorder to, here, the selection and design of a pharmaceutically effective counter. In terms of molecular diseases it is an unique achievement: e.g. even after 70 years there is still no effective counter to the sickle-haemoglobin mutation. The paper outlines and clearly describes careful and painstaking studies. The deductions and conclusions are data-based and frank (no hype!). There are some caveats that do not affect the strength of the paper but to which the authors should respond and modify as appropriate.
Over the period of thirty years there has inevitably been relevant refinements as to the underlying mechanism of polymerisation. There is now totally persuasive evidence that the fundamental defect is the aberration of the bonding centred on Glu342 that pinions strand 5a in place and hence guides the final incorporation of the C-terminus into the body of the antitrypsin molecule and subsequently hinders its aberrant release. The supporting evidence is summarised in the paper of Huang et al (JBC 2016: 291,15674) on the 'Molecular mechanism of Z alpha-1-antitrypsin deficiency', which includes the crystal structure of Z antitrypsin. It should be cited. The Huang paper in no way pre-empts the current submission and indeed greatly strengthens it, almost as a preliminary study. The two papers are complementary, with satisfying agreements as to the drug-binding site. The very real advance in the submitted paper being the confirmation structurally of what is otherwise a deduced binding-site and in the design of a ligand that preferentially binds to the Z variant rather than the M (normal wild-type).
The submitted paper describes new and important findings but it presents these in what is in places a dated context. The findings here precisely fit with the consensus mechanism that is now supported by a whole body of studies in the field. It is important to get this right which could be achieved with some minor rewriting. As it is, the Discussion can be misleading, as in 'The finding that GSK716 mediates its action by binding to a cryptic pocket implies that intrahepatic polymers form a near native or native conformation, rather than a more extended intermediate.' This is not so, and should be removed. We are looking at the stabilisation of equilibria, be it in the partially folded form or the fully-folded but labile aberrant native form.
An outstanding finding in the submitted paper is in the determination of the structure of the antitrypsin-GSK17 complex. Although this structure seemed instinctively correct the data was succinct and a backup expert crystallographic opinion was sought, as follows.
'The crystallography is almost certainly fine, judging from the overall statistics and the resolution, but there are a few things that should be explained better.
When a structure is deposited at the PDB, the authors receive a validation report that is intended for potential reviewers. This should be provided with the manuscript to allow referees to assess the quality of the structural work.
The authors have left some blanks in Table 1. There is a line for Resolution in the Data collection section, but it's blank. Similarly, in the Refinement section the line for Water under "No. atoms" is blank, and both lines for Protein and Water under B-factors are blank. If numbers are given in these two subsections for protein and water, they should also be given for the ligand. In particular, it's important to be able to compare the mean B-factor of the ligand with that of the protein.
Whenever an important part of a structural paper is the binding of a ligand, it is accepted practice to provide a figure showing electron density for the ligand. Ideally, this should be unbiased electron density, computed either from a difference map before the ligand was added or after omitting the ligand and carrying out some refinement.
One comment: the paper reports that the structure was solved by molecular replacement, using a model that had originally been obtained from PDB entry 2qug. Since 2qug seems to be isomorphous to this structure, it would have been simpler to carry out rigid-body refinement than to use molecular replacement, which will give a structure with a different choice of origin and symmetry copy; this makes structure comparison less convenient to users of the structures than the rigid-body refinement option.'

The authors should respond to these points and the Editor/Journal note the desirability of making PDB validation reports available to reviewing referees.

Referee #2 (Comments on Novelty/Model System for Author):

The models are well-suited to this proof of principle analysis.

Referee #2 (Remarks for Author):

The paper presents an analysis of a small molecule corrector of AAT folding that interdicts polymerization of Z-type AAT. The candidate molecule was identified by high throughput screening from a large library. The paper from a distinguished group of investigators is well-written and compelling. The curious findings are that there is no reduction in intrahepatic polymers despite a 7-fold increase in circulating Z-type AAT and that the lower doses (10 and 30 mg/kg) exerted the same effect as the 100 mg/kg dose despite low free durg levels. The authors do offer conjecture to explain these findings. They also offer an explanation as to how the opposing effects of GSK716 to decrease serine proteinase inhibition of AAT vs. to enhance secretion of Z-type AAT might result in net benefit. Of course, definitively understanding the net effect of GSK716 will require further study. Within the context of a proof-of-principle analysis, including examining the effects in mice, the paper offers an important contribution.

Referee #3 (Comments on Novelty/Model System for Author):

Cell line models are used to demonstrate that the lead compound, GSK716, reduces the intracellular accumulation of polymers and increases secretion of Z-AAT. Similar results were obtained using the PiZ mouse model. However, this study did not provide convincing evidence demonstrating the beneficial effects of abolished polymerization and increased secretion of Z-AAT in the cell- or mouse-models. In Fig. 2f, the authors show that the treatment with GSK716 modestly improved the viability of Z-AAT expressing cells following a "second insult" namely, tunicamycin treatment. However, M-AAT expressing cells + GSK716 control was notably missing. Furthermore, it is unclear why 13-15 week old, PiZ hemizygous mice were used in this study. At this age, the authors themselves report that there were no signs of liver fibrosis. Use of mice at an age with early signs of fibrosis may have been more informative in determining whether GSK716 treatment could prevent progression or reverse existing fibrosis.

Referee #3 (Remarks for Author):

It is widely accepted that the Z-mutant of AAT accumulates as polymers within the endoplasmic reticulum of hepatocytes and this is responsible for the liver disease associated with severe AAT-deficiency. It is also widely accepted that the accumulation of Z-AAT in the liver, significantly impairs secretion of AAT into the plasma. The reduced circulating AAT is thought to be responsible, as least in part, for the lung disease associated with AAT-deficiency. As such, a compound that abolishes Z-AAT polymer formation and consequently enhances secretion of AAT would have beneficial effects on both the liver and lung diseases associated with AAT-deficiency. In an effort to identify such a compound, the authors conducted a screen and identified a strong lead compound, GSK716, that blocked Z-polymer accumulation and increased secretion of Z-AAT in their cell line

models. Further studies performed in mice showed an apparent increase in secretion as determined by an increase in circulating Z-AAT in the plasma. Unfortunately, the compound had no effect on preventing the accumulation of Z-AAT polymers in the mouse hepatocytes.

Although the in vitro data on the lead compound's ability to prevent polymerization is impressive, it is unclear, from this study, whether the decrease in accumulation of Z-AAT is actually beneficial to the cells in anyway. The authors tried to answer this question by generating a tunicamycin survival curve (Fig. 2f). Interestingly, GSK716 treatment modestly improved viability of Z-expressing cells, however, an important control, notably M-expressing cells treated with GSK716 was missing. Further studies are required to address this important question.

In some instances, there is inadequate information, in the figure legends and in the methods, to adequately understand how the experiments were conducted and what the results actually mean. The authors indicated that GSK716 "increased the secretion of Z a1-antitrypsin approximately 3-fold compared to vehicle control (mean pEC50 6.2 {plus minus} 0.23; n = 74) (Fig. 2b)," however, it is unclear how they came to that conclusion from the data provided. Fig. 2b show 100% secretion. Fig. 2d shows 3-fold secretion. What does 100% secretion mean? What does 3-fold secretion mean? More information should be included to assist the reader in determining the significance of the data.

The authors then provide data in the PiZ mouse model showing that GSK716 treatment significantly increased plasma levels of Z-AAT. They concluded that this was due to increased secretion of Z-AAT from the liver, however, it is unclear whether this is actually the case since the amount of Z-AAT in the hepatocytes remained unchanged in control- and GSK716-treated mice.

Although, GSK716 failed to alter Z-AAT polymer accumulation in the liver, one could argue that the 7-fold increase in secretion of Z-AAT would be beneficial to the lung disease associated with AAT-deficiency. Unfortunately, GSK716 binds to Z-AAT in such a way that it abolishes the anti-proteinase activity of Z-AAT. This is a critically important as it implies that increasing secretion of Z-AAT has no apparent physiological benefits.

The authors' conclusion that "This study provides proof-of-principle that 'mutation ameliorating' small molecules are a viable approach to treat protein conformational diseases" Is not supported by the data presented in this manuscript.

Minor points:

GSK716 is sometimes referred to as GSK'716A. Is this a different analog or a typo?

Fig. 2g, bottom right, should be "extracellular" not intracellular.

Referee #1 (Remarks for Author):

This paper represents a triumphant progress, led by the first author over a period of nearly 30 years, from the initial proposal of a conformational basis for a common inherited disorder to, here, the selection and design of a pharmaceutically effective counter. In terms of molecular diseases it is an unique achievement: e.g. even after 70 years there is still no effective counter to the sickle-haemoglobin mutation. The paper outlines and clearly describes careful and painstaking studies. The deductions and conclusions are data-based and frank (no hype!). There are some caveats that do not affect the strength of the paper but to which the authors should respond and modify as appropriate.

We are very grateful to the reviewer for his/her supportive comments.

Over the period of thirty years there has inevitably been relevant refinements as to the underlying mechanism of polymerisation. There is now totally persuasive evidence that the fundamental defect is the aberration of the bonding centred on Glu342 that pinions strand 5a in place and hence guides the final incorporation of the C-terminus into the body of the antitrypsin molecule and subsequently hinders its aberrant release. The supporting evidence is summarised in the paper of Huang et al (JBC 2016: 291,15674) on the 'Molecular mechanism of Z alpha-1-antitrypsin deficiency', which includes the crystal structure of Z antitrypsin. It should be cited. The Huang paper in no way pre-empts the current submission and indeed greatly strengthens it, almost as a preliminary study. The two papers are complementary, with satisfying agreements as to the drug-binding site. The very real advance in the submitted paper being the confirmation structurally of what is otherwise a deduced binding-site and in the design of a ligand that preferentially binds to the Z variant rather than the M (normal wild-type).

The submitted paper describes new and important findings but it presents these in what is in places a dated context. The findings here precisely fit with the consensus mechanism that is now supported by a whole body of studies in the field. It is important to get this right which could be achieved with some minor rewriting. As it is, the Discussion can be misleading, as in 'The finding that GSK716 mediates its action by binding to a cryptic pocket implies that intrahepatic polymers form a near native or native conformation, rather than a more extended intermediate.' This is not so, and should be removed. We are looking at the stabilisation of equilibria, be it in the partially folded form or the fully-folded but labile aberrant native form.

We fully agree with the reviewer and have added the reference to Huang *et al*, 2016. Indeed, our group has recently used cryo-EM to characterise isolated polymers from the liver tissue of Z α_1-AT homozygotes (Glu342Lys) who had undergone liver transplantation. The data show that the inter-subunit linkage of Z α_1-AT is best explained by a C-terminal domain swap between molecules of α_1-AT (Faull *et al*, 2020). These data are consistent with a head-to-tail arrangement of subunits in heat-induced polymers revealed by a complex with the non-Z-selective antibody 2H2 (Laffranchi *et al*, 2020). This is in keeping with the view expressed by the reviewer. We have removed 'The finding that GSK716 mediates its action by binding to a cryptic pocket implies that intrahepatic polymers form a near native or native conformation, rather than a more extended intermediate.'

We have changed the introduction to read ‘Polymerisation from this state involves insertion of the RCL into β-sheet A with a domain-swap of the C-terminal region providing the inter-subunit linkage (Faull *et al*, 2020; Huang *et al*, 2016; Laffranchi *et al*, 2020). The resulting polymer is deposited within hepatocytes.

The text ‘polymerises from a near-native conformation late in the folding pathway (Motamedi-Shad *et al*, 2016; Ordóñez *et al*, 2015) and therefore’ has been deleted from the results section. ‘The finding that GSK716 mediates its action by binding to a cryptic pocket implies that intrahepatic polymers form from a near-native or native conformation, rather than a more extended intermediate’ has been deleted from the discussion and replaced with ‘GSK716 stabilises the partially folded α_1-antitrypsin or the fully-folded but labile aberrant native form.’

An outstanding finding in the submitted paper is in the determination of the structure of the antitrypsin-GSK17 complex. Although this structure seemed instinctively correct the data was succinct and a backup expert crystallographic opinion was sought, as follows. 'The crystallography is almost certainly fine, judging from the overall statistics and the resolution, but there are a few things that should be explained better. When a structure is deposited at the PDB, the authors receive a validation report that is intended for potential reviewers. This should be provided with the manuscript to allow referees to assess the quality of the structural work.

We have provided the validation report from the PDB submission (D_1292111290_val-report-full_P1.pdf). The PDB entry is 7EAL. Unfortunately, the submission has been given the wrong title. We have asked for this to be corrected but there is currently a technical issue with the platform. The data in the PDB are correct.

The authors have left some blanks in Table 1. There is a line for Resolution in the Data collection section, but it's blank. Similarly, in the Refinement section the line for Water under "No. atoms" is blank, and both lines for Protein and Water under B-factors are blank. If numbers are given in these two subsections for protein and water, they should also be given for the ligand. In particular, it's important to be able to compare the mean B-factor of the ligand with that of the protein.

We are sorry that this was missed from the paper. The full Table has been included in the paper and is reproduced below.

Table 1. Data collection and refinement statistics

PDBFILE	7AEL
Temperature	100K
Wavelength	0.9763
Resolution range	55.05 - 1.76 (1.823 - 1.76)
Space group	C 1 2 1
Unit cell	113.954 39.592 90.517 90 104.961 90
Total reflections	127818 (12581)
Unique reflections	38772 (3853)
Multiplicity	3.3 (3.3)
Completeness (%)	99.2 (99.2)
Mean I/sigma(I)	19.61 (2.19)
Wilson B-factor	32.95
R-merge	0.02941 (0.5007)
R-meas	0.03523 (0.5991)
CC1/2	0.999 (0.805)
CC*	1 (0.944)
Reflections used in refinement	38771 (3853)
Reflections used for R-free	1908 (178)
R-work	0.1969 (0.3065)
R-free	0.2259 (0.3204)
CC(work)	0.958 (0.739)
CC(free)	0.943 (0.699)
Number of non-hydrogen atoms	3189
macromolecules	2868
ligands	32
Protein residues	357
RMS(bonds)	0.005
RMS(angles)	1.02
Ramachandran favored (%)	99
Ramachandran allowed (%)	1.1
Ramachandran outliers (%)	0
Rotamer outliers (%)	0.62
Clashscore	0.52
Average B-factor	47.79
macromolecules	46.77
ligands	36.49
solvent	59.23

Statistics for the highest-resolution shell are shown in parentheses

Whenever an important part of a structural paper is the binding of a ligand, it is accepted practice to provide a figure showing electron density for the ligand. Ideally, this should be unbiased electron density, computed either from a difference map before the ligand was added or after omitting the ligand and carrying out some refinement.

The OMIT density maps for the ligand is shown below and reproduced in Figs. 3F and G.

OMIT (Fo-Fc)map	OMIT (2Fo-Fc)map

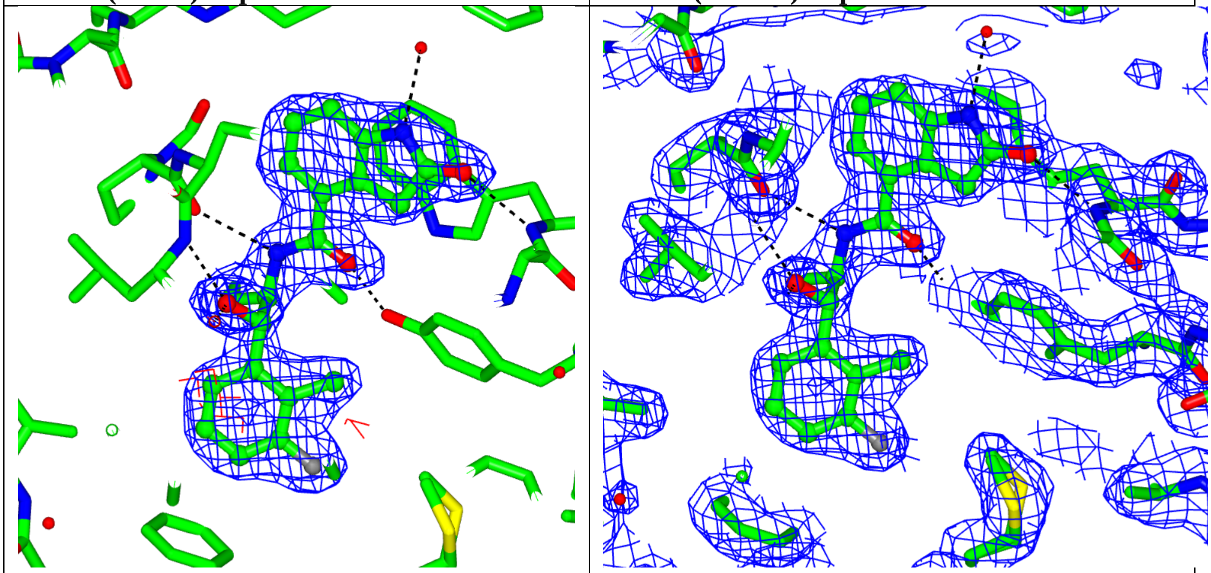

±3.0sigma (blue/red)	+1.0 sigma (blue)

One comment: the paper reports that the structure was solved by molecular replacement, using a model that had originally been obtained from PDB entry 2qug. Since 2qug seems to be isomorphous to this structure, it would have been simpler to carry out rigid-body refinement than to use molecular replacement, which will give a structure with a different choice of origin and symmetry copy; this makes structure comparison less convenient to users of the structures than the rigid-body refinement option.'

We have ensured the deposited structure, 7AEL.pdb, shares the same choice of origin as 2qug.pdb

The authors should respond to these points and the Editor/Journal note the desirability of making PDB validation reports available to reviewing referees.

The validation report has been included with the resubmitted manuscript.

Referee #2 (Comments on Novelty/Model System for Author):

The models are well-suited to this proof of principle analysis.

Referee #2 (Remarks for Author):
The paper presents an analysis of a small molecule corrector of AAT folding that interdicts polymerization of Z-type AAT. The candidate molecule was identified by high throughput screening from a large library. The paper from a distinguished group of investigators is well-written and compelling. The curious findings are that there is no reduction in intrahepatic polymers despite a 7-fold increase in circulating Z-type AAT and that the lower doses (10 and 30 mg/kg) exerted the same effect as the 100 mg/kg dose despite low free durg levels. The authors do offer conjecture to explain these findings. They also offer an explanation as

to how the opposing effects of GSK716 to decrease serine proteinase inhibition of AAT vs. to enhance secretion of Z-type AAT might result in net benefit. Of course, definitively understanding the net effect of GSK716 will require further study. Within the context of a proof-of-principle analysis, including examining the effects in mice, the paper offers an important contribution.

We are very grateful to the reviewer for his/her supportive comments

Referee #3 (Comments on Novelty/Model System for Author):

Cell line models are used to demonstrate that the lead compound, GSK716, reduces the intracellular accumulation of polymers and increases secretion of Z-AAT. Similar results were obtained using the PiZ mouse model. However, this study did not provide convincing evidence demonstrating the beneficial effects of abolished polymerization and increased secretion of Z-AAT in the cell- or mouse-models. In Fig. 2f, the authors show that the treatment with GSK716 modestly improved the viability of Z-AAT expressing cells following a "second insult" namely, tunicamycin treatment. However, M-AAT expressing cells + GSK716 control was notably missing.

We are grateful to the reviewer for his/her comments. We have repeated the experiment with M α_1-AT expressing cells + GSK716. The new figure is reproduced below along with the figure legend. Cells expressing M α_1-AT were more resistant to tunicamycin than cells expressing Z α_1-AT. 716 increased the resistance of Z α_1-AT cells to tunicamycin to that of M α_1-AT. It had no effect on the survival of cells expressing M α_1-AT. This is included as revised Fig. 2F.

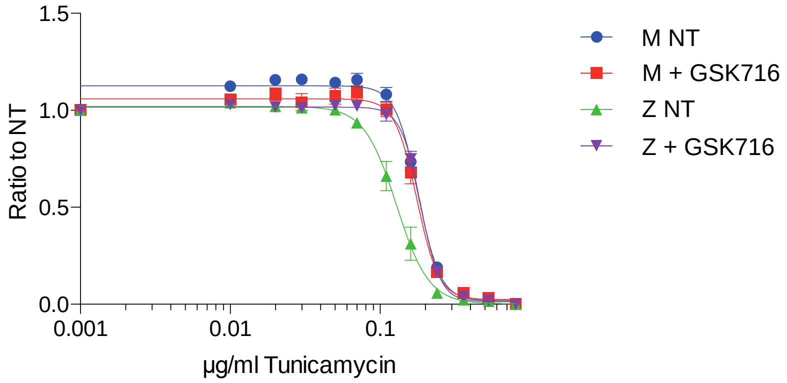

(F) CHO inducible cells expressing either wildtype M or Z α_1-antitrypsin were induced with 0.5µg/mL doxycycline and treated with 10µM GSK716 or with 0.1% DMSO vehicle (NT, not treated). After induction for 48 h, cells were treated with various doses of tunicamycin for 36 h. Cell viability was measured by Cell Counting Kit-8. The results are shown as mean±SEM, n=4.

Furthermore, it is unclear why 13-15 week old, PiZ hemizygous mice were used in this study. At this age, the authors themselves report that there were no signs of liver fibrosis. Use of mice at an age with early signs of fibrosis may have been more informative in determining whether GSK716 treatment could prevent progression or reverse existing fibrosis.

The mouse model has been used widely by the community to test novel therapies for α_1-AT deficiency, two of which have now progressed to evaluation in humans (Guo *et al*. J Clin Invest. 2014;124:251-61; Hidvegi T *et al*, Science. 2010;329:229-32; Pastore *et al*,. Hepatology. 2017; 65:1865-1874). The primary aim of the mouse experiments was to assess target engagement and in particular whether GSK716 could increase the secretion of Z α_1-AT. Having shown this, we then looked to evaluate whether there was an effect on hepatic inclusions. We selected younger, and hemizygous, rather than older mice, as our longevity studies showed that circulating levels of Z α_1-AT increase with age (to levels much higher than seen in patients) and these artificially high levels may act as a high affinity sink sequestering drug and preventing bioavailability at the site of action in the liver. Further, older mice have larger α_1-AT inclusions that may be more difficult to reverse and any changes would be more difficult to detect than in younger animals. As the reviewer comments, the conclusion from our studies is that GSK716 did not reduce PAS positive inclusions, following testing in animals where the compound had the best chance of impacting them, and would also therefore be unlikely to reverse fibrosis.

The following text has been added to the results section 'Younger hemizygous, rather than older mice, were selected as our longevity studies showed that circulating levels of Z α_1-antitrypsin increase with age (to levels much higher than seen in patients) and these artificially high levels may act as a high affinity sink sequestering drug and preventing bioavailability at the site of action in the liver. Further, older mice have larger α_1-antitrypsin inclusions that may be more difficult to reverse and any changes would be more difficult to detect than in younger animals.'

Referee #3 (Remarks for Author):

It is widely accepted that the Z-mutant of AAT accumulates as polymers within the endoplasmic reticulum of hepatocytes and this is responsible for the liver disease associated with severe AAT-deficiency. It is also widely accepted that the accumulation of Z-AAT in the liver, significantly impairs secretion of AAT into the plasma. The reduced circulating AAT is thought to be responsible, as least in part, for the lung disease associated with AAT-deficiency. As such, a compound that abolishes Z-AAT polymer formation and consequently enhances secretion of AAT would have beneficial effects on both the liver and lung diseases associated with AAT-deficiency. In an effort to identify such a compound, the authors conducted a screen and identified a strong lead compound, GSK716, that blocked Z-polymer accumulation and increased secretion of Z-AAT in their cell line models. Further studies performed in mice showed an apparent increase in secretion as determined by an increase in circulating Z-AAT in the plasma. Unfortunately, the compound had no effect on preventing the accumulation of Z-AAT polymers in the mouse hepatocytes.
Although the in vitro data on the lead compound's ability to prevent polymerization is impressive, it is unclear, from this study, whether the decrease in accumulation of Z-AAT is actually beneficial to the cells in anyway. The authors tried to answer this question by generating a tunicamycin survival curve (Fig. 2f). Interestingly, GSK716 treatment modestly improved viability of Z-expressing cells, however, an important control, notably M-expressing cells treated with GSK716 was missing. Further studies are required to address this important question.

We have repeated the experiment in Fig 2F with M α_1-AT expressing cells + GSK716 as described in the section above

In some instances, there is inadequate information, in the figure legends and in the methods, to adequately understand how the experiments were conducted and what the results actually mean. The authors indicated that GSK716 "increased the secretion of Z a1-

antitrypsin approximately 3-fold compared to vehicle control (mean pEC50 6.2 {plus minus} 0.23; n = 74) (Fig. 2b)," however, it is unclear how they came to that conclusion from the data provided. Fig. 2b show 100% secretion. Fig. 2d shows 3-fold secretion. What does 100% secretion mean? What does 3-fold secretion mean? More information should be included to assist the reader in determining the significance of the data.

We thank the reviewer for raising this point. We have changed the legend to Fig 2B and 2D to make it clear that data in Fig. 2B (CHO-TET-ON-Z-A1AT cell assay) are normalised, using vehicle and a control compound (from the GSK716 series) at saturating concentration as high/low controls, as is standard practise for screening assays. The actual concentrations of α_1-AT in the supernatants were not determined in the same experiments, and we have removed the comment about 3-fold increase of secretion in this context. However, in the iPSC-hepatocyte assays (Fig 2D), calibration curves were used to determine the levels of α_1-AT in the supernatant in each individual experiment, therefore the fold increase induced by GSK716, compared with vehicle control, could be calculated and is shown in Fig 2D. The statement in the text is consistent with these data.

The text has been changed to read (new text in red). In comparison with controls, GSK716 completely blocked the intracellular formation of Z α_1-antitrypsin polymers, as measured by staining with the 2C1 anti-Z α_1-antitrypsin polymer monoclonal antibody (mean pIC50 = 6.3 ± 0.23; n = 71) (Figs. 2A, B). It also increased the secretion of Z α_1-antitrypsin (mean pEC50 6.2 ± 0.23; n = 74) (Fig. 2B). Similar potency between the effects on secretion and polymerisation was observed throughout members of the lead series supporting the hypothesis that these effects are caused by the same pharmacological mode of action. GSK716 had a similar effect on the secretion and polymerisation of constitutively expressed Z α_1-antitrypsin in iPSC-derived human hepatocytes with the ZZ α_1-antitrypsin genotype (Yusa *et al*, 2011). It inhibited polymerisation and increased secretion with a mean pIC50 of 6.4 ± 0.45 (n = 16) and mean pEC50 of 6.5 ± 0.37 (n = 14), respectively, inducing an approximately 3-fold increase of secreted levels of Z α_1-antitrypsin (Figs 2C, D).

Figure 2. GSK716 inhibits polymerisation of Z α_1-antitrypsin in cell models of disease. (A) GSK716 was added to CHO-TET-ON-Z-A1AT cells (Ordóñez *et al.*, 2013) with simultaneous induction of Z α_1-antitrypsin expression using doxycycline, and polymer load was quantified with the 2C1 monoclonal antibody that is specific to pathological polymers of α_1-antitrypsin (Miranda *et al.*, 2010). The parent cell line that did not express Z α_1-antitrypsin provided a negative control. GSK716 completely prevented intracellular polymer formation. (B) Quantification of immunostained CHO-TET-ON-Z-A1AT cells showed that GSK716 reduced intracellular polymer formation and increased the secretion of Z α_1-antitrypsin in a dose-dependent manner with similar potencies. Data were normalised to vehicle and a control compound from the GSK716 series at saturating concentration. (C) GSK716 was administered to iPSC-derived-hepatocytes and (D) inhibited polymerisation and increased secretion with a similar potency. It induced an approximately 3-fold increase in secreted levels of Z α_1-antitrypsin compared with vehicle control. This was apparent even after polymers had been allowed to form.

We have also amended the abstract to read 'The lead compound blocks Z α_1-antitrypsin polymerisation *in vitro*, reduces intracellular polymerisation and increases the secretion of Z α_1-antitrypsin three-fold in an iPSC model of disease' and have corrected the text in the Discussion.

The authors then provide data in the PiZ mouse model showing that GSK716 treatment significantly increased plasma levels of Z-AAT. They concluded that this was due to increased secretion of Z-AAT from the liver, however, it is unclear whether this is actually the

case since the amount of Z-AAT in the hepatocytes remained unchanged in control- and GSK716-treated mice.

The conclusion that the increased secretion is from the liver is based on: (i) the pulse chase experiments that showed GSK716 increased the secretion of Z α_1-AT from CHO cells and iPSC derived hepatocytes (Fig. 3). (ii) treatment with GSK716 increased the monomer measured in liver homogenate by approx. 4-fold in keeping with the changes seen in blood and (iii) liver is the only tissue that can produce sufficient α_1-AT to cause a 7 fold increase in circulating protein.

Although, GSK716 failed to alter Z-AAT polymer accumulation in the liver, one could argue that the 7-fold increase in secretion of Z-AAT would be beneficial to the lung disease associated with AAT-deficiency. Unfortunately, GSK716 binds to Z-AAT in such a way that it abolishes the anti-proteinase activity of Z-AAT. This is a critically important as it implies that increasing secretion of Z-AAT has no apparent physiological benefits.

The authors' conclusion that "This study provides proof-of-principle that 'mutation ameliorating' small molecules are a viable approach to treat protein conformational diseases" Is not supported by the data presented in this manuscript.

We are grateful to the reviewer for raising this issue. The statement referred to our finding that GSK716 can bind to a site close to the mutation in Z-AAT and change its polymerisation behaviour to approximate that of M-AAT. We apologise for the confusion and have amended this sentence in the abstract and the impact section to read ‘can block the aberrant polymerisation that underlies Z α_1-antitrypsin deficiency’.

Minor points:

GSK716 is sometimes referred to as GSK'716A. Is this a different analog or a typo?

GSK716 and GSK’716 are the same. We have amended Fig. 2 to only refer to GSK716.

Fig. 2G, bottom right, should be "extracellular" not intracellular.

This has been corrected in the revised manuscript.

1st Dec 2020

Dear Prof. Lomas,

Thank you for the submission of your revised manuscript to EMBO Molecular Medicine. I am pleased to inform you that we will be able to accept your manuscript pending the following final amendments:

1) Figures: Please remove all figures from the main manuscript file and upload 1 file per figure. Figure legends and EV figure legend should stay in the manuscript text file.
2) Tables: Please upload Table EV1 as individual file. The legend for Table EV1 should be removed from the manuscript text file and added to the Table EV1 file in a separate tab.
3) In the main manuscript file, please do the following:
- Correct/answer the track changes suggested by our data editors by working from the attached/uploaded document.

***** Reviewer's comments *****

Referee #1 (Remarks for Author):

I have carefully reviewed the paper and now unequivocally recommend publication.

Referee #3 (Remarks for Author):

The authors have addressed the concerns raised in the initial review. As such I recommend it for publication in the EMBO Molecular Medicine.

2nd Authors' Response to Reviewers 8th Dec 2020

The authors performed the requested changes.

10th Dec 2020

Dear Prof. Lomas,

We are pleased to inform you that your manuscript is accepted for publication.

EMBO PRESS

YOU MUST COMPLETE ALL CELLS WITH A PINK BACKGROUND ➔

PLEASE NOTE THAT THIS CHECKLIST WILL BE PUBLISHED ALONGSIDE YOUR PAPER

Corresponding Author Name:	Jogender Tushir-Singh
Journal Submitted to:	EMBO Molecular Medicine
Manuscript Number:	EMM-2020-12716

Reporting Checklist For Life Sciences Articles (Rev. June 2017)

This checklist is used to ensure good reporting standards and to improve the reproducibility of published results. These guidelines are consistent with the Principles and Guidelines for Reporting Preclinical Research issued by the NIH in 2014. Please follow the journal's authorship guidelines in preparing your manuscript.

A- Figures
1. Data
The data shown in figures should satisfy the following conditions:

- ➔ the data were obtained and processed according to the field's best practice and are presented to reflect the results of the experiments in an accurate and unbiased manner.
- ➔ figure panels include only data points, measurements or observations that can be compared to each other in a scientifically meaningful way.
- ➔ graphs include clearly labeled error bars for independent experiments and sample sizes. Unless justified, error bars should not be shown for technical replicates.
- ➔ if n< 5, the individual data points from each experiment should be plotted and any statistical test employed should be justified
- ➔ Source Data should be included to report the data underlying graphs. Please follow the guidelines set out in the author ship guidelines on Data Presentation.

2. Captions

Each figure caption should contain the following information, for each panel where they are relevant:

- ➔ a specification of the experimental system investigated (eg cell line, species name).
- ➔ the assay(s) and method(s) used to carry out the reported observations and measurements
- ➔ an explicit mention of the biological and chemical entity(ies) that are being measured.
- ➔ an explicit mention of the biological and chemical entity(ies) that are altered/varied/perturbed in a controlled manner.

- ➔ the exact sample size (n) for each experimental group/condition, given as a number, not a range;
- ➔ a description of the sample collection allowing the reader to understand whether the samples represent technical or biological replicates (including how many animals, litters, cultures, etc.).
- ➔ a statement of how many times the experiment shown was independently replicated in the laboratory.
- ➔ definitions of statistical methods and measures:
  - common tests, such as t-test (please specify whether paired vs. unpaired), simple χ2 tests, Wilcoxon and Mann-Whitney tests, can be unambiguously identified by name only, but more complex techniques should be described in the methods section;
  - are tests one-sided or two-sided?
  - are there adjustments for multiple comparisons?
  - exact statistical test results, e.g., P values = x but not P values < x;
  - definition of 'center values' as median or average;
  - definition of error bars as s.d. or s.e.m.

Any descriptions too long for the figure legend should be included in the methods section and/or with the source data.

In the pink boxes below, please ensure that the answers to the following questions are reported in the manuscript itself. Every question should be answered. If the question is not relevant to your research, please write NA (non applicable). We encourage you to include a specific subsection in the methods section for statistics, reagents, animal models and human subjects.

USEFUL LINKS FOR COMPLETING THIS FORM

http://www.antibodypedia.com
http://1degreebio.org
http://www.equator-network.org/reporting-guidelines/improving-bioscience-research-repor
http://grants.nih.gov/grants/olaw/olaw.htm
http://www.mrc.ac.uk/Ourresearch/Ethicsresearchguidance/Useofanimals/index.htm
http://ClinicalTrials.gov
http://www.consort-statement.org
http://www.consort-statement.org/checklists/view/32-consort/66-title
http://www.equator-network.org/reporting-guidelines/reporting-recommendations-for-tumo
http://datadryad.org
http://figshare.com
http://www.ncbi.nlm.nih.gov/gap
http://www.ebi.ac.uk/ega
http://biomodels.net/
http://biomodels.net/miriam/
http://jjj.biochem.sun.ac.za
http://oba.od.nih.gov/biosecurity/biosecurity_documents.html
http://www.selectagents.gov/

B- Statistics and general methods

Please fill out these boxes ➔ (Do not worry if you cannot see all your text once you press return)

1.a. How was the sample size chosen to ensure adequate power to detect a pre-specified effect size?	There are 3-6 animals/group. Some groups where size-matched 1 or 2 animals are shown (such as Figure 6A, 6I), the experiments were repeated 3 times. Thus a minimum of 3 animals were used except for IHC studies.
1.b. For animal studies, include a statement about sample size estimate even if no statistical methods were used.	Statistical methods were used for all experiments except IHC studies, where one representative tumor from the treatment group was analyzed.
2. Describe inclusion/exclusion criteria if samples or animals were excluded from the analysis. Were the criteria pre-established?	No, animals were not excluded from the analysis
3. Were any steps taken to minimize the effects of subjective bias when allocating animals/samples to treatment (e.g. randomization procedure)? If yes, please describe.	Tumor-bearing animals prior to treatment of different antibodies (which are being compared to each other) were randomly selected. To minimize bias, if animals had an initial smaller tumor size, they were equally distributed to each treatment group. There was no subjective bias in animal selection.
For animal studies, include a statement about randomization even if no randomization was used.	Tumor-bearing animals prior to particular treatment were randomly selected. There was no subjective bias in animal selection.
4.a. Were any steps taken to minimize the effects of subjective bias during group allocation or/and when assessing results (e.g. blinding of the investigator)? If yes please describe.	To minimize bias, if there were animals that had an initial smaller tumor size, they were equally distributed to each treatment group.
4.b. For animal studies, include a statement about blinding even if no blinding was done	Yes
5. For every figure, are statistical tests justified as appropriate?	Yes
Do the data meet the assumptions of the tests (e.g., normal distribution)? Describe any methods used to assess it.	Yes Non parametric tests were used.
Is there an estimate of variation within each group of data?	Yes

Is the variance similar between the groups that are being statistically compared?	No For experiments where treatment results in significant differences in tumor size, the follow-up analysis was carried out by size match to avoid variation before statistical comparison.

C- Reagents

6. To show that antibodies were profiled for use in the system under study (assay and species), provide a citation, catalog number and/or clone number, supplementary information or reference to an antibody validation profile. e.g., Antibodypedia (see link list at top right), 1DegreeBio (see link list at top right).	All DR5 agonist antibodies were produced using CHO cell expression system. The procedure for antibody production, surface binding and function has been tested and has previously been described (Shivange et al., 2018, Cancer Cell). All antibodies were diluted in 1X PBS (pH 7.2) to appropriate concentration (as indicated in figures or corresponding figure legends) prior to cellular and animal treatments.
7. Identify the source of cell lines and report if they were recently authenticated (e.g., by STR profiling) and tested for mycoplasma contamination.	All cells in our laboratory are tested for being negtive for mycoplasma

* for all hyperlinks, please see the table at the top right of the document

D- Animal Models

8. Report species, strain, gender, age of animals and genetic modification status where applicable. Please detail housing and husbandry conditions and the source of animals.	6 to 8 weeks-old (Age), 20-25-gram (Weight), both male and female (Sex) mice were used for tumor xenograft generation, in vivo efficacy studies, imaging studies, TIL isolation studies as described in the text. Following mice stains were used: C56BL/6 (Jackson laboratories) Balb/C (Jackson laboratories), immunodeficient Balb/c derived athymic Nude Foxn1nu/Foxn1+ (Envigo) and NOD.Cg Prkdcscid Il2rgtm1Wjl/SzJ is also called as NSG mice. All animal procedures were conducted in accordance with the University of Virginia Institutional Animal Care and Use Committee (IACUC) and (DoD ACURO) approved protocols and conform to the relevant regulatory standards.
9. For experiments involving live vertebrates, include a statement of compliance with ethical regulations and identify the committee(s) approving the experiments.	All animal procedures were conducted under the accordance of University of Virginia Institutional Animal Care and Use Committee (IACUC) and (DoD ACURO) approved protocols and conform to the relevant regulatory standards. All animals were handled in strict accordance with good animal practice as per University regulations.
10. We recommend consulting the ARRIVE guidelines (see link list at top right) (PLoS Biol. 8(6), e1000412, 2010) to ensure that other relevant aspects of animal studies are adequately reported. See author guidelines, under 'Reporting Guidelines'. See also: NIH (see link list at top right) and MRC (see link list at top right) recommendations. Please confirm compliance.	Yes

E- Human Subjects

11. Identify the committee(s) approving the study protocol.	Not Applicable
12. Include a statement confirming that informed consent was obtained from all subjects and that the experiments conformed to the principles set out in the WMA Declaration of Helsinki and the Department of Health and Human Services Belmont Report.	Not Applicable
13. For publication of patient photos, include a statement confirming that consent to publish was obtained.	Not Applicable
14. Report any restrictions on the availability (and/or on the use) of human data or samples.	Not Applicable
15. Report the clinical trial registration number (at ClinicalTrials.gov or equivalent), where applicable.	Not Applicable
16. For phase II and III randomized controlled trials, please refer to the CONSORT flow diagram (see link list at top right) and submit the CONSORT checklist (see link list at top right) with your submission. See author guidelines, under 'Reporting Guidelines'. Please confirm you have submitted this list.	Not Applicable
17. For tumor marker prognostic studies, we recommend that you follow the REMARK reporting guidelines (see link list at top right). See author guidelines, under 'Reporting Guidelines'. Please confirm you have followed these guidelines.	Not Applicable

F- Data Accessibility

18: Provide a "Data Availability" section at the end of the Materials & Methods, listing the accession codes for data generated in this study and deposited in a public database (e.g. RNA-Seq data: Gene Expression Omnibus GSE39462, Proteomics data: PRIDE PXD000208 etc.) Please refer to our author guidelines for 'Data Deposition'.<br><br>Data deposition in a public repository is mandatory for:<br>a. Protein, DNA and RNA sequences<br>b. Macromolecular structures<br>c. Crystallographic data for small molecules<br>d. Functional genomics data<br>e. Proteomics and molecular interactions	Not Applicable as NO large OMICs data was generated.
19. Deposition is strongly recommended for any datasets that are central and integral to the study; please consider the journal's data policy. If no structured public repository exists for a given data type, we encourage the provision of datasets in the manuscript as a Supplementary Document (see author guidelines under 'Expanded View' or in unstructured repositories such as Dryad (see link list at top right) or Figshare (see link list at top right).	Not Applicable
20. Access to human clinical and genomic datasets should be provided with as few restrictions as possible while respecting ethical obligations to the patients and relevant medical and legal issues. If practically possible and compatible with the individual consent agreement used in the study, such data should be deposited in one of the major public access-controlled repositories such as dbGAP (see link list at top right) or EGA (see link list at top right).	Not Applicable
21. Computational models that are central and integral to a study should be shared without restrictions and provided in a machine-readable form. The relevant accession numbers or links should be provided. When possible, standardized format (SBML, CellML) should be used instead of scripts (e.g. MATLAB). Authors are strongly encouraged to follow the MIRIAM guidelines (see link list at top right) and deposit their model in a public database such as Biomodels (see link list at top right) or JWS Online (see link list at top right). If computer source code is provided with the paper, it should be deposited in a public repository or included in supplementary information.	Not Applicable

G- Dual use research of concern

22. Could your study fall under dual use research restrictions? Please check biosecurity documents (see link list at top right) and list of select agents and toxins (APHIS/CDC) (see link list at top right). According to our biosecurity guidelines, provide a statement only if it could.	Not Applicable
